# In situ continuous hydrogen-bonded engineering for intrinsically stretchable and healable high-mobility polymer semiconductors

**DOI:** 10.1126/sciadv.adq0171

**Published:** 2024-10-02

**Authors:** Haoguo Yue, Ying Wang, Shaochuan Luo, Junfeng Guo, Jun Jin, Gongxi Li, Zhihao Meng, Lei Zhang, Dongshan Zhou, Yonggang Zhen, Wenping Hu

**Affiliations:** ^1^ Advanced Innovation Center for Soft Matter Science and Engineering, State Key Laboratory of Organic-Inorganic Composites, Beijing University of Chemical Technology, Beijing 100029, China.; ^2^ Wuhan National Laboratory for Optoelectronics, Huazhong University of Science and Technology, Wuhan 430074, China.; ^3^ State Key Laboratory of Fine Chemicals, School of Chemical Engineering, Dalian University of Technology, Dalian 116024, China.; ^4^ Department of Polymer Science and Engineering, State Key Laboratory of Coordination Chemistry, Key Laboratory of High Performance Polymer Material and Technology, School of Chemistry and Chemical Engineering, Nanjing University, Nanjing 210023, China.; ^5^ MOE Key Laboratory of Organic Integrated Circuits & Tianjin Key Laboratory of Molecular Optoelectronic Sciences, Department of Chemistry, School of Sciences, Tianjin University, Tianjin 300072, China.

## Abstract

As a key component for wearable electronics, intrinsically stretchable and healable semiconducting polymers are scarce because carrier mobility is often reduced with increasing stretchability and self-healability. Here, we combine stepwise polymerization and thermal conversion to introduce in situ continuous hydrogen bonding sites in a polymer backbone without breaking the conjugation or introducing bulky softer side chains, benefiting the intrachain and interchain charge transport. We demonstrate that a regular sequence structure facilitated the formation of big nanofibers with a high degree of aggregation, providing the loose and porous thin film with simultaneously improved charge transport, stretchability, and self-healability. The mobility of damaged devices can be recovered to 81% after a healing treatment. Fully stretchable transistor based on the designed polymer exhibited a greatly enhanced mobility up to 1.08 square centimeters per volt per second under 100% strain, which is an unprecedented value and constitutes a major step for the development of intrinsically stretchable and healable semiconducting polymers.

## Teaser

Intrinsically stretchable and healable polymer semiconductors were achieved without conjugation breakers or bulky side chains.

## INTRODUCTION

Stretchable electronic devices have found potential applications in health monitoring, artificial skins, and implantable bioelectronics (*1*–*5*). Unfortunately, it has been a long-standing problem that the accumulation of uncontrolled damages by repeated wear and tear, accidental cutting, or scratching in the process of actual use would lead to an attenuation of the device performance and a reduction in the service life (*6*–*9*). In terms of this issue, intrinsically stretchable polymer semiconductors that have the self-healing ability to repair inflicted damages are becoming increasingly important in electronic devices due to their low cost, amenability to large-area printing, and high-density device manufacturing (*10*–*13*). High stretchability and self-healing properties can be achieved by incorporation of dynamic noncovalent bonds through side-chain engineering or backbone engineering (*14*–*16*). The dynamic bonds can easily be broken to facilitate strain energy dissipation, making the materials more tolerant to strain and mechanical stimuli, which can be reformed to recover the initial mechanical properties. However, charge carrier mobility is often reduced with increasing stretchability and self-healability, making a big challenge for the development of stretchable and self-healing polymer semiconductors. Therefore, there are only a few studies on the stretchable and self-healable polymer semiconductors to date (*14*–*17*).

**Fig. 1.**
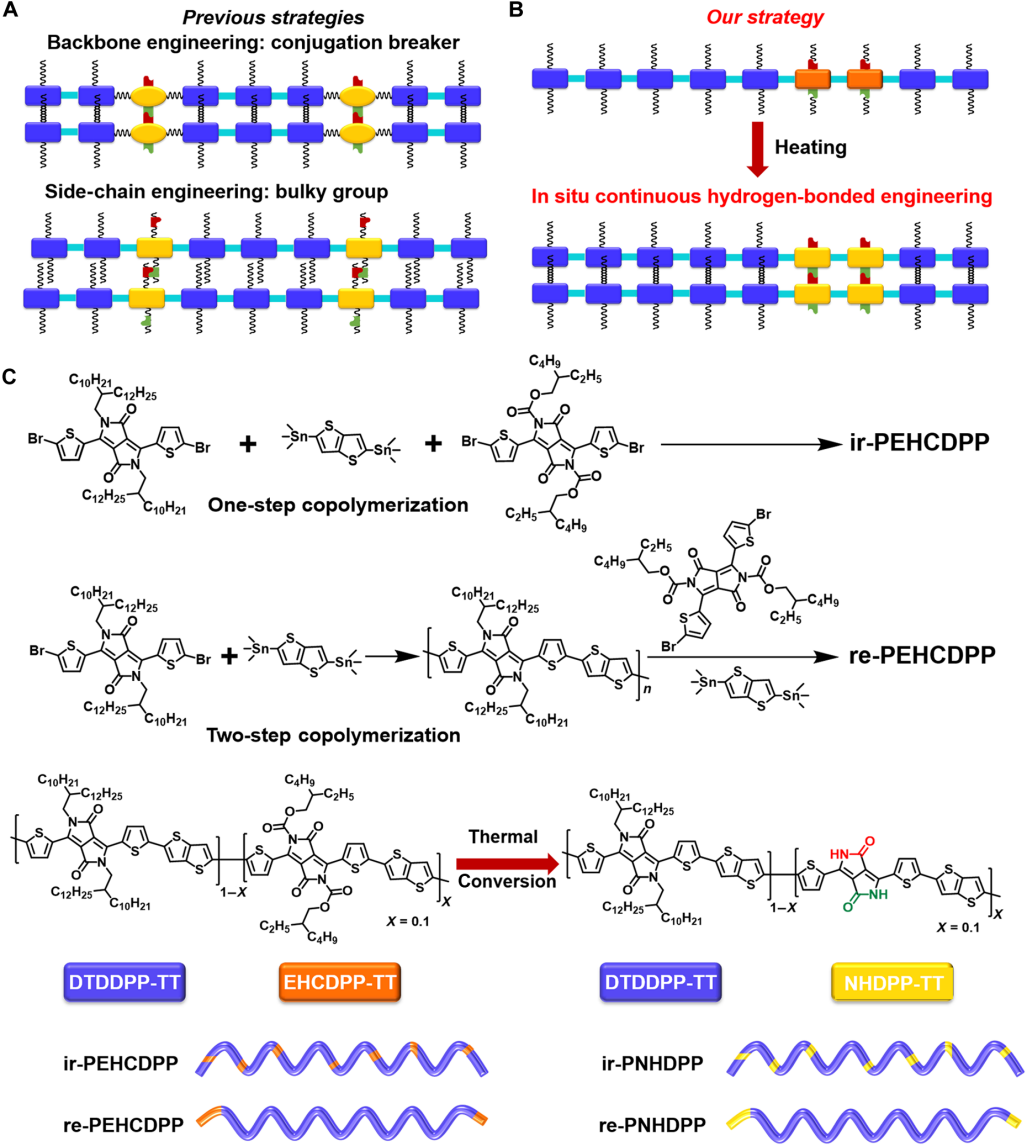
Design strategies of intrinsically stretchable polymers. (**A**) Schematics of methods for preparing intrinsically stretchable polymers reported in the literature (*14*,*15*). (**B**) Schematics of methods for preparing intrinsically stretchable polymers designed by this work. (**C**) In situ formation of ir-PNHDPP and re-PNHDPP polymers by thermal conversion of ir-PEHCDPP and re-PEHCDPP, which were synthesized by one-step and two-step Stille coupling copolymerization, respectively.

Among the different types of noncovalent interactions, hydrogen bonds are particularly investigated for skin-inspired electronics owing to their spontaneous formation and healing ability (*14*, *15*, *18*–*20*). For example, Oh *et al.* (*14*) demonstrated a backbone engineering approach by incorporating a pyridine dicarboxamide moiety as a conjugation-break spacer into diketopyrrolopyrrole (DPP)–based polymers, which showed a hole mobility of greater than 0.1 cm^2^ V^−1^ s^−1^ while being stretched to 100% elongation and recovered nearly to initial mobility upon healing for the damaged films. Lee *et al.* (*15*) presented self-healing DPP-based polymers with urethane side chains, providing moderate H-bonding and sufficient solubility. The thin films maintained the carrier mobility of ~0.03 cm^2^ V^−1^ s^−1^ under 100% strain, featuring self-healing properties with partial recovery of electrical properties after treatment. The introduction of flexible noncovalent conjugation breakers into the polymer backbone adversely affected the intrachain charge transport, while the incorporation of longer and softer side chains with dynamic bonds distinctly weakened the interchain charge transport (Fig. 1A) (*13*, *21*). Moreover, the dynamic bonds are generally introduced randomly and discontinuously as a conjugation breaker or side-chain group by one-step copolymerization. On the contrary, no studies have been investigated on the incorporation of the continuous dynamic bonds in a polymer backbone in terms of charge transport, stretchability, and healability.

Herein, we combine stepwise polymerization and thermal conversion to introduce continuous hydrogen bonding sites in a DPP polymer backbone without breaking the conjugation or introducing bulky softer side chains, thus facilitating the intrachain and interchain charge transport while maintaining excellent stretchability and healability (Fig. 1B). We partially replaced alky chains with the thermal removable 2-ethylhexoxycarbonyl (EHC) group to obtain relatively regular and irregular DPP polymers (re-PEHCDPP and ir-PEHCDPP) by changing the feeding sequences of relative monomers (Fig. 1C). After removal of EHC side chains by thermal annealing, both polymers were converted to the polymers with liberated N–H groups (re-PNHDPP and ir-PNHDPP), which can interact with the C=O groups on the DPP units of the neighboring polymer chains to form intermolecular hydrogen bonds of N–H⋯O=C (*22*, *23*). Compared with ir-PNHDPP, the re-PNHDPP polymer, having continuous *N*-alkyl chains and N–H sites, formed a loose, porous, and fibrous thin film with a high degree of aggregation, long-range ordering, and increased chain dynamics. Although most intrinsically stretchable polymer semiconductors showed degraded mobilities under strain (*24*, *25*), the polymer re-PNHDPP exhibited 1.8-fold increased hole mobility under 75% strain, which is the highest enhancement to date (*13*, *15*, *26*). The field-effect mobility of damaged devices can be recovered to 81% after a solvent and thermal healing treatment. Furthermore, we synthesized block polymer bl-PNHDPP with a more regular sequence structure and fabricated the fully stretched transistors based on bl-PNHDPP, showing greatly enhanced mobilities up to 1.27 and 1.08 cm^2^ V^−1^ s^−1^ under 75 and 100% strain, respectively, to our knowledge, which are the unprecedented values for intrinsically stretchable and healable semiconducting polymers and among the highest mobilities for intrinsically stretchable polymer semiconductors (*13*–*15*, *27*–*29*). Although long-range ordering is supposed to be detrimental to dissipate applied mechanical stress (*13*, *28*), both good stretchability and high mobility were realized in our designed polymers. The long-range ordering contributed to the improvement of charge transport, while the loose and porous structure of fibrous thin films with increased chain dynamics was favorable to notably enhance stretchability. Moreover, continuous hydrogen bonds were able to undergo a strain energy dissipation or healing mechanism through breakage or reformation of bonds. Therefore, in situ continuous hydrogen-bonded engineering can be used as a powerful strategy to develop stretchable and healable high-performance polymer semiconductors.

## RESULTS

### Material synthesis and characterization

In this work, we designed and synthesized regular and irregular EHC-modified DPP polymers (re-PEHCDPP and ir-PEHCDPP) by conventional Stille coupling polycondensation among 3,6-bis-(5-bromo-thiophen-2-yl)-2,5-bis(2-decyltetradecyl)-1,4-dioxo-pyrrolo[3,4-*c*]pyrrole (DTDDPP; 0.9 equiv), 3,6-bis(5-bromo-thiophen-2-yl)-2,5-bis(2-ethylhexyl-carboxylate)-1,4-dioxo-pyrrolo[3,4-*c*]pyrrole (EHCDPP; 0.1 equiv), and 2,5-bis(trimethylstannyl)thieno[3,2-*b*]thiophene (TT; 1 equiv) (Fig. 1C and figs. S1 and S2). ir-PEHCDPP was obtained by mixing the above monomers together at the same time (one-step copolymerization), while re-PEHCDPP was achieved by subsequent addition of EHCDPP (0.1 equiv) and TT (0.1 equiv) into the reaction mixture of DTDDPP (0.9 equiv) and TT (0.9 equiv) that was polymerized (two-step copolymerization). A 10 mol % (0.1 equiv) solution of EHCDPP was selected because 10 mol % hydrogen bonding units usually showed good balance between stretchability and mobility (*14*, *24*, *30*). As for ir-PEHCDPP, the chain of DTDDPP-TT was constantly disrupted by EHCDPP-TT because the three monomers underwent the polymerization simultaneously, providing a less regular sequence structure in the polymer backbone. As for re-PEHCDPP, first, the polymerization between DTDDPP and TT proceeded to give the longer chain of DTDDPP-TT. Second, the followed EHCDPP and TT tended to polymerize to form the longer chain of EHCDPP-TT, rather than copolymerized with DTDDPP-TT due to lower reactivity of the long chain of DTDDPP-TT. Last, the long chain of DTDDPP-TT coupled with the long chain of EHCDPP-TT with the same reactivity, affording the more regular sequence structure in the polymer backbone. We selected EHC side chains as thermal removable groups because the EHC unit endows the polymers with the high temperature stability and great solubility (*22*), which allowed the Stille coupling to carry out smoothly without removal of EHC side chains and made the device fabrication easy by a solution-processed method. The different comonomers usually disrupt short-range aggregation, resulting in reduced carrier mobilities (*28*, *31*). In our molecular design, we considered that the same backbone in comonomers EHCDPP and DTDDPP was favorable to maintain short-range aggregation. The molecular weight and dispersity were summarized in the Supplementary Materials (table S1). Introducing EHCDPP-TT segments was expected to show different ^1^H nuclear magnetic resonance (NMR) spectra and decrease the C element content. As revealed by high-temperature ^1^H NMR spectroscopy, the peak at 4.38 parts per million (ppm) can be ascribed to the –CH_2_ groups of EHC side chains connected to the ester O atoms (EHCDPP) by comparison of that of the relative group for the starting material EHCDPP (fig. S3). The ratios of EHCDPP in re-PEHCDPP and ir-PEHCDPP were determined as 9.45 and 10.64%, respectively, by elemental analysis (C, H, N, and S elements) as summarized in the Supplementary Materials (table S2).

The EHC side chains in re-PEHCDPP and ir-PEHCDPP were removed smoothly by thermal annealing of both thin films at 200°C for 30 min, leading to the conversion into re-PNHDPP and ir-PNHDPP, respectively, with complete insolubility in a common organic solvent such as chloroform and chlorobenzene (figs. S4 to S6). Both re-PNHDPP and the reference NHDPP-TT oligomer exhibited a broad shoulder peak at 11 to 13 ppm, which can be ascribed to the continuous hydrogen bonding sites, while the shoulder peak cannot be observed in ir-PNHDPP (fig. S7). From Fourier transform infrared (FTIR) spectroscopy (fig. S8), the bands in the region of 2800 to 3000 cm^−1^ assigned to alkyl chains weakened after annealing, indicating that the EHC moieties were eliminated from the polymer backbone. Furthermore, two sharp absorption bands at ~3440 and ~3233 cm^−1^ suggest the formation of N–H bonds and hydrogen-bonded networks. We also conducted in situ FTIR spectroscopy measurement for re-PNHDPP at 200°C (fig. S9); all the bands remained almost unchanged after the sample was heated for 8 hours compared with that for 30 min, indicating that the thermal conversion proceeded entirely for 30 min for the thin film state. Thermogravimetric analysis was used to investigate the thermal properties of re-PEHCDPP and ir-PEHCDPP (fig. S10). Both polymers started to lose weight at ~200°C but showed much higher temperatures for total removal of EHC chains, probably because of the more difficult evaporation of the decomposed side-chain fragments in the bulk samples than in the thin films (*22*, *32*). re-PEHCDPP and ir-PEHCDPP showed a weight loss of 2.85 and 2.89%, respectively, deriving the ratio of EHC side chains as 9.79 and 9.93%, which are in good agreement with the results from elemental analysis.

The ultraviolet-visible (UV-vis)/near-infrared (NIR) absorption spectra were measured to examine the optical and aggregation properties of re-PEHCDPP and ir-PEHCDPP, displaying typical π-π* transition (300 to 500 nm) and intramolecular charge transfer bands (550 to 1000 nm) both in solutions and in thin films (fig. S11) (*33*–*35*). The polymer thin films showed broader absorption profiles and a pronounced red shift than their corresponding solutions, which could be related to effective solid-state packing effects. The lower energy 0-0 vibronic peak was typically attributed to interchain stacking (*36*). As polymer semiconductors often form aggregates, comparing the intensity of 0-1 (*I*_0-1_) and 0-0 (*I*_0-0_) vibronic peaks can provide information regarding the relative degree of aggregation (*37*). Both EHC-removed polymers ir-PNHDPP and re-PNHDPP displayed similar absorption profiles with a small red shift as well as increased *I*_0-0_/*I*_0-1_ values relative to the pristine film, because of the formation of “head-to-tail” arrangement and the enhanced degree of aggregation caused by intermolecular hydrogen bonding in thin films (*38*). Compared with ir-PNHDPP, re-PNHDPP showed an obvious bathochromic-shifted absorption spectrum with enhanced *I*_0-0_/*I*_0-1_ values, suggesting that the regular arrangement with more continuous *N*-alkyl and N–H segments resulted in a stronger interchain stacking state that is beneficial to efficient charge carrier transport.

### Improved mechanical properties and self-healing ability without compromising mobility

**Fig. 2.**
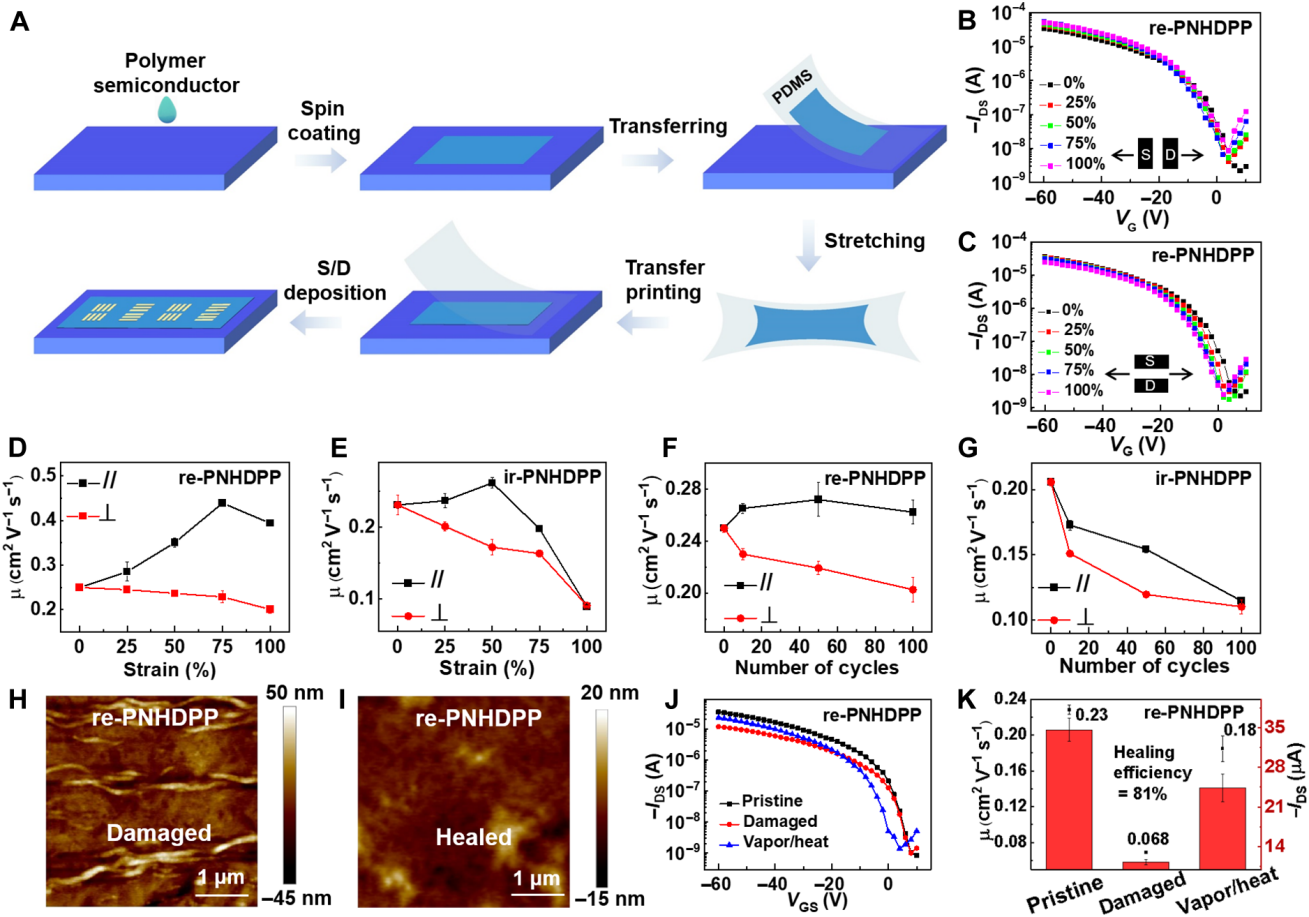
Charge transport properties of two stretchable polymers re-PNHDPP and ir-PNHDPP. (**A**) Illustration of the fabrication process for OFETs with the stretched thin films. S/D, source/drain. (**B**) Transfer curves of the re-PNHDPP film at different strains along the charge transport direction. (**C**) Transfer curves of the re-PNHDPP film at different strains along the perpendicular to charge transport direction. (**D**) Field-effect mobility as a function of various strains of re-PNHDPP. (**E**) Field-effect mobility as a function of various strains of ir-PNHDPP. (**F**) Field-effect mobility versus the number of stretching cycles of re-PNHDPP. (**G**) Field-effect mobility versus the number of stretching cycles of ir-PNHDPP. (**H**) AFM images for the damaged film of re-PNHDPP. (**I**) AFM images for the healed film of re-PNHDPP. (**J**) Transfer curves of the pristine, damaged, and healed re-PNHDPP film. (**K**) Field-effect mobility and on-current of the pristine, damaged, and healed re-PNHDPP film.

First, the electrical and mechanical properties of re-PEHCDPP and ir-PEHCDPP were measured. The charge transport features of re-PEHCDPP and ir-PEHCDPP thin films were evaluated using organic field-effect transistors (OFETs) with the bottom-gate/top-contact (BGTC) architecture. Typical transfer (fig. S12) and output curves (fig. S13) of both polymers exhibited the p-type semiconducting characteristics. The EHC-removed polymers showed significantly enhanced average mobilities, from 0.05 to 0.25 cm^2^ V^−1^ s^−1^ for re-PNHDPP and from 0.02 to 0.21 cm^2^ V^−1^ s^−1^ for ir-PNHDPP (fig. S14). *V*_GS_-dependent mobility was extracted for re-PNHDPP and ir-PNHDPP films. For the re-PNHDPP film, negligible gate voltage dependence of mobility was observed. Although the *V*_GS_-dependent mobility plot showed a peak mobility at low *V*_GS_ for ir-PNHDPP, we did not extract the mobility at low voltages to avoid the overestimation of the mobility (fig. S15). To investigate the charge transport behavior under mechanical deformation, the films of both polymers spin-coated on the octadecyltrichlorosilane (OTS)/SiO_2_ substrates were peeled off, stretched at varying strains, and then laminated onto SiO_2_/Si substrates to form the BGTC device for electrical measurements (Fig. 2A) (*14*, *39*). As shown in Fig. 2, both polymers exhibited different trends in mobility when stretched. The device performances were summarized in tables S3 and S4. The fidelity factors (*r*) were calculated to be over 88% for all the devices, suggesting that the mobility values extracted from the traditional transistor equations are reliable (*40*). For re-PNHDPP thin films (Fig. 2, B and D, and fig. S16), the mobility reached a maximum value (0.44 cm^2^ V^−1^ s^−1^) under 75% strain with 1.8-fold increase and then began to decrease slightly as much as 1.6-fold under 100% strain relative to that of 0% strain in the parallel direction (//). The improvement in mobility is highly impressive under 75 or 100% strain, outperforming other reported intrinsically stretchable polymer semiconductors usually with a pronounced decrease in mobility under strain (*14*, *16*, *24*, *41*). The relatively low clockwise hysteresis was observed at all tested strains, which is also observed normally in most polymers (*42*, *43*), can be attributed to the charge trapping at the interface (fig. S17). For ir-PNHDPP thin films (Fig. 2E and figs. S18A and S19), in comparison, the mobility showed a maximum value under 50% strain with only 1.1-fold increase and then decreased sharply by a factor of 2.6 under 100% strain relative to that of 0% strain. re-PNHDPP–based devices maintained decent mobility with a small decrease by 20% under 100% strain along the perpendicular stretching direction (⊥), while ir-PNHDPP–based devices exhibited distinctly degraded mobility with a reduction of 61% under the same conditions (Fig. 2, C to E, and figs. S18B and S19). In addition, we tested the environmental long-term stability for re-PNHDPP and ir-PNHDPP films stored in air for 18 months. Compared to the initial devices, slight changes in both threshold voltage shifts and carrier mobilities were observed, indicating the stability of the films (figs. S20 to S23).

Second, rigorous repeated stretching cycle tests were conducted by stretching both re-PNHDPP and ir-PNHDPP thin films for up to 100 cycles of repeated stretching with 25% strain (Fig. 2, F and G, and figs. S24 to S26). The device performances were summarized in tables S5 and S6. Because most practical applications require effective operation only for applied strains of 20 to 30% (*14*), along the parallel stretching direction, the mobility of re-PNHDPP initially increased at 25% strain after 10 stretching-releasing cycles and then remained stable with the increase in the number of cycles. Notably, the charge mobility at 25% strain after 100 stretching cycles was still higher than that of the original unstretched film. In contrast, the mobility of ir-PNHDPP continuously decreased with the increase in the number of cycles at 25% strain and lastly dropped markedly by 44% when subjected to 100 stretching cycles. Along the perpendicular stretching direction, the re-PNHDPP thin films showed higher durability with only a 19% decrease in mobility observed for 100 cycles up to 25% strain while the ir-PNHDPP thin films revealed a 47% reduction under the same conditions.

Third, the healing properties of re-PNHDPP and ir-PNHDPP polymer films were investigated, considering the reconstruction of hydrogen bonds in the DPP polymer backbone after breakage. The films were intentionally damaged to form nanocracks with a strain of 100% for re-PNHDPP and 50% for ir-PNHDPP up to 10 cycles and then posttreatments via heat and solvent annealing were used to promote polymer chain movement. The treatments were divided into two steps and repeated three times: (i) exposure of the damaged samples under chloroform vapor for 10 min (ii) and then thermal annealing of the damaged samples at 120°C for 10 min. After mechanical deformation, dense nanocracks in the thin films appeared as revealed by atomic force microscopy (AFM) analysis. When both solvent and thermal annealing were applied, a complete disappearance of nanocracks for re-PNHDPP films and a great reduction in size and density of the nanocracks for ir-PNHDPP films were observed, respectively (Fig. 2, H and I, and fig. S27, A and B). Furthermore, the electrical properties of films were evaluated (Fig. 2, J and K, and fig. S27, C and D). When damaged, the mobility abruptly decreased by a factor of 3.4 or 2.2 for re-PNHDPP or ir-PNHDPP, respectively. After healing treatment, the hole mobility and average on-current were greatly recovered for re-PNHDPP, with the mobility being 81% of that in the pristine thin film. In contrast, the hole mobility for ir-PNHDPP was recovered to 73% and the on-current was only slightly recovered relative to the pristine film. We demonstrate that the introduction of dynamic noncovalent bonds among the rigid conjugated backbone is possible to afford good self-healing properties. Overall, these results indicated that the polymer re-PNHDPP with more continuous and concentrated hydrogen bonds exhibited much more efficient healing ability when subjected to mechanical deformation and nanocrack formation.

### Controlled film microstructures by introducing hydrogen bonds in the polymer backbone

**Fig. 3.**
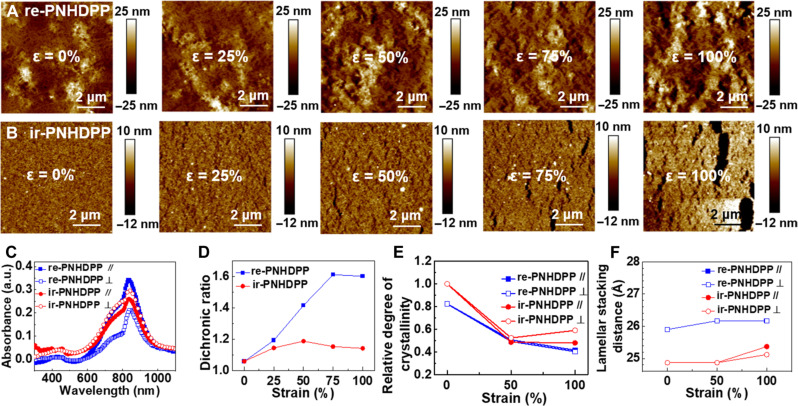
Micromorphologies, chain alignments, and crystallinity properties of re-PNHDPP and ir-PNHDPP thin films when stretched. (**A**) AFM images of re-PNHDPP films under different tensile strains. (**B**) AFM images of ir-PNHDPP films under different tensile strains. (**C**) Polarized UV-vis absorption spectra of re-PNHDPP and ir-PNHDPP films at 75% strain. a.u., arbitrary units. (**D**) Dichroic ratio values of re-PNHDPP and ir-PNHDPP films. (**E**) Changes of the rDoC extracted from the peak (200) of re-PNHDPP and ir-PNHDPP films under strain. (**F**) Lamellar stacking distances of re-PNHDPP and ir-PNHDPP films under different tensile strains.

To gain deep insight into our polymer design achieving both high mobility and great mechanical properties, we first performed AFM and optical microscope measurements to characterize the surface morphology and the formation of cracks of the polymer thin films (Fig. 3 and figs. S27 to S31). There was no noticeable change in the AFM images of re-PEHCDPP before and after annealing; both of which showed a loose, porous, and fibrous thin film, although we observed that the nanofiber network became looser after annealing for ir-PEHCDPP (fig. S32). Impressively, we observed that re-PNHDPP (Fig. 3A) formed large coiled nanofibers with a diameter of ~50 nm, which is much larger than that of ir-PEHCDPP (Fig. 3B) and other reported intrinsically stretchable semiconducting polymers (*13*, *14*, *21*, *28*, *29*), plausibly due to the strong interchain stacking state as revealed by the increased *I*_0-0_/*I*_0-1_ value from UV-vis/NIR absorption spectra. re-PNHDPP thin films exhibited relatively small cracks perpendicular to the stretching direction at 100% strain (fig. S28). In contrast, ir-PNHDPP thin films showed some small cracks under only 50% strain (fig. S29), which were further propagated to dissipate strain energy with increasing strain, indicative of a lower crack onset strain.

Next, we used polarized optical microscopy to confirm the polymer chain alignment for thin films when stretched. We found that the unstretched film was isotropic, while 75% stretched re-PNHDPP thin films displayed prominent anisotropic light transmission when rotated 45° with respect to the light polarization direction, indicating the obvious anisotropy under strain (fig. S33). We further measured the degree of polymer chain alignment under strain using polarized UV-vis/NIR spectroscopy (Fig. 3C). The dichroic ratio initially increased due to the strain-induced chain alignment and then decreased owing to the crack formation (Fig. 3D) (*14*, *44*, *45*). re-PNHDPP thin films showed a higher dichroic ratio, indicating a higher degree of polymer chain alignment. In addition, the dichroic ratio of re-PNHDPP began to decrease under 100% strain, implying that the crack formation occurred at greater strain compared with ir-PNHDPP. The stretched films of both polymers showed red-shifted absorption bands in the regions of 300 to 500 and 550 to 1000 nm, indicating that the polymer backbones became straighter and more planar under strain (fig. S34, A to D). *I*_0-0_/*I*_0-1_ values for both polymers increased when stretched. re-PNHDPP showed a much larger *I*_0-0_/*I*_0-1_ value than ir-PNHDPP under strain, suggesting a higher degree of interchain stacking state (fig. S34, E and F). The highest carrier mobility of re-PNHDPP or ir-PNHDPP was achieved along the parallel stretching direction at the highest degree of polymer chain alignment when the dichroic ratio reached a maximum value under 75 or 50% strain, respectively. The trends in crack onset strain and dichroic ratio for both polymers under strain corresponded well with the relative changes in mobilities, demonstrating that the polymer chain alignment and continuous fibrous network are highly important to the charge transport behavior.

We further investigated the influence of the sequence structure on the chain dynamics of the re-PNHDPP or ir-PNHDPP film by the alternating current chip calorimetry (*46*). The glass transition temperature (*T*_g_) values of the polymer backbone were determined to be −6.0° and 2.5°C for re-PNHDPP to ir-PNHDPP, respectively (fig. S35), suggesting the viscoelasticity for two polymers at room temperature and increased chain dynamics for re-PNHDPP. In addition, the tensile modulus of re-PNHDPP (0.52 GPa) was measured to be 20% lower than that of ir-PNHDPP (0.65 GPa) by AFM nanomechanical mapping (fig. S36), indicating the higher film deformability and ductility for re-PNHDPP.

To understand how microstructures of the polymer thin films were affected by the different sequence structures in the polymer backbone of re-PNHDPP and ir-PNHDPP, we performed grazing incidence wide-angle x-ray scattering (GIWAXS) measurement. The data revealed that the crystallinity of two polymers (re-PNHDPP and ir-PNHDPP) was markedly improved in terms of the relative degree of crystallinity (rDoC) compared to the polymers before thermal annealing (figs. S37 and S38). Both of the polymers dominantly formed an edge-on orientation with similar π-π stacking and remained the same under strain (fig. S39). The rDoC with strain was calculated based on the (200) lamellar stacking peak in the two-dimensional GIWAXS diffractograms of polymer thin films (Fig. 3E and figs. S40 and S41). re-PNHDPP has lower crystallinity than ir-PNHDPP in terms of the rDoC despite its higher aggregation degree and larger nanofibers. We observed that the rDoC of ir-PNHDPP decreased with strain from 0 to 50% and then plateaued, suggesting the partial energy dissipation through breakage of the crystalline regions and further crack formation at a higher strain. In contrast, the rDoC of re-PNHDPP decreased steadily and slowly with increasing strain from 0 to 100%. We characterized the change of crystalline domains by examining the lamellar stacking distance extracted from (100) peaks. The lamellar distance of re-PNHDPP was slightly larger than that of ir-PNHDPP. re-PNHDPP exhibited the less change under strain (Fig. 3F), which suggested that the re-PNHDPP crystalline domains underwent the smaller deformation due to the lower rDoC. Using the Scherrer equation, the coherence length of crystalline domains for re-PNHDPP was calculated to be 123.5 Å, which was larger than that of ir-PNHDPP (110.8 Å) (table S7) (*13*).

We then carried out cryo–transmission electron microscopy (TEM) measurement to study the film nanostructures and aggregation states of re-PNHDPP and ir-PNHDPP. We observed that re-PNHDPP had a small number of local aggregates with large sizes while ir-PNHDPP had a large number of local aggregates with small sizes (figs. S42 and S43). Large lamellar stacking fringes with a length up to 90 nm and a width up to 21 nm were clearly observed with neighboring disordered domains in the case of re-PNHDPP (fig. S44). The lamellar distance of re-PNHDPP was in the range of 22.2 to 23.5 Å, which was relatively smaller than that derived from GIWAXS, revealing the average values for the thin film. As for ir-PNHDPP, small lamellar stacking fringes were formed with a length of ≤11 nm, a width of ≤13 nm, and a lamellar distance of 20.8 to 22.0 Å (fig. S45). Compared with ir-PNHDPP, re-PNHDPP had a higher degree of long-range ordering and wider lamellar distance, corresponding well with those of GIWAXS. The continuous *N*-alkyl segments of re-PNHDPP were favorable to interact with the same segments of adjacent polymer chains and then self-assembled into large lamellar stacking domains with a high degree of aggregation. In contrast, the inconsecutive and disordered *N*-alkyl segments for ir-PNHDPP with an irregular sequence structure were difficult to form efficient interchain stacking and then afforded small crystalline domains with a low degree of aggregation. Film microstructures are generally controlled to prevent the generation of large crystalline domains, which are supposed to be detrimental to dissipate applied mechanical stress (*13*, *28*). Very unexpectedly, good stretchability was realized despite the presence of long-range ordering, probably because of the formation of loose and porous thin films with big-size nanofibers having continuous hydrogen bonding and increased chain dynamics.

### The proposed mechanism for good stretchability, healability, and carrier mobility

**Fig. 4.**
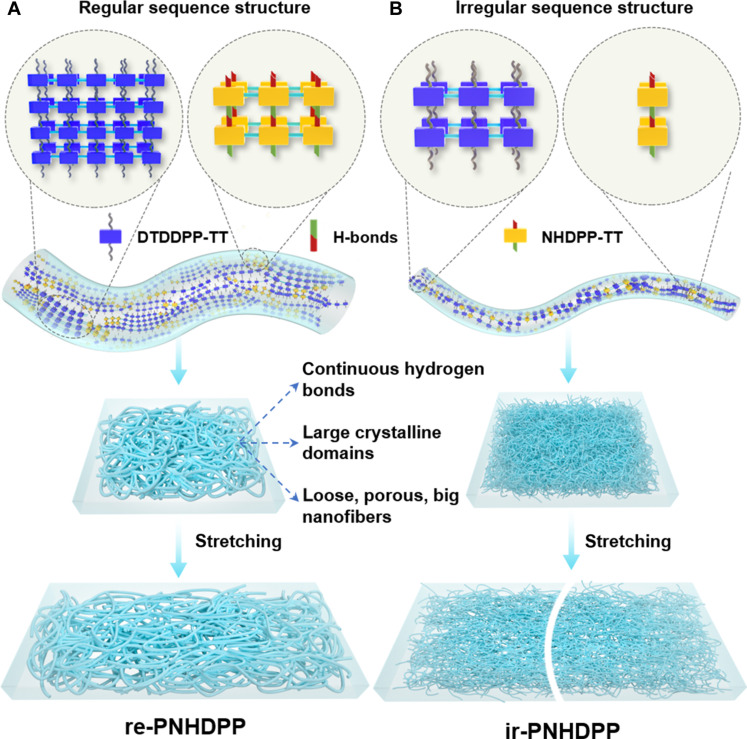
Deformation mechanism of re-PNHDPP and ir-PNHDPP. (**A**) Schematic diagrams illustrating mechanisms for energy dissipation during strain in the loose and porous film of re-PNHDPP consisting of big nanofibers with continuous hydrogen bonds and long-range ordering. (**B**) Schematic diagrams illustrating mechanisms for energy dissipation during strain in the dense and less porous fibrous film of ir-PNHDPP consisting of small nanofibers with incontinuous hydrogen bonds and short-range ordering.

We hypothesized that the regular sequence structure in the polymer backbone was highly important to achieve excellent carrier mobility, stretchability, and healability simultaneously. The regular sequence structure not only facilitated the interactions of continuous *N*-alkyl segments but also promoted hydrogen bonding of continuous N–H segments, resulting into efficient interchain stacking. In contrast, the incontinuous N–H segments were not conducive to intermolecular hydrogen bonding owing to the large steric hinderance effects of alkyl chains neighboring to N–H units. Compared with ir-PNHDPP, re-PNHDPP had a high degree of aggregation and long-range ordering (Fig. 4), improving distinctly the charge transport behavior. It is noteworthy that the lower crystallinity, higher chain dynamics, and lower tensile modulus, revealed by GIWAXS, *T*_g_ measurements, and AFM nanomechanical mapping, contributed to the improvement of stretchability for re-PNHDPP. Moreover, the sufficient number of consecutive intermolecular hydrogen bonds was favorable to enhance substantially stretchability and self-healing ability. The mechanism for strain energy dissipation can be proposed as follows. At the onset of stretching, the energy was dissipated by stretching coiled nanofibers. The loose, porous, and fibrous thin film for re-PNHDPP had a much larger void volume to allow the nanofiber to elongate or contract along the parallel or perpendicular stretching direction, respectively, giving higher stretchability under strain (*47*). The extended polymer chains were effectively aligned as revealed by polarized absorption spectroscopy, facilitating both intrachain and interchain charge transport. Next, as more strain stress was applied, energy was dissipated by the gradual disruption of hydrogen bonding (*14*). In the case of ir-PNHDPP, the dense, less porous thin film had a limited void volume to prevent nanofibers to deform freely under strain. Moreover, the less continuous NHDPP-TT units had weaker hydrogen bonds, thus showing lower stretchability. When damaged, re-PNHDPP thin films were supposed to be healed in a higher efficiency considering that the more continuous and concentrated hydrogen bonds had high probability to be reformed due to larger noncovalent interactions, which were consistent with the self-healing ability measurements.

### Further optimization of polymer structures and fabrication of fully stretchable transistors

**Fig. 5.**
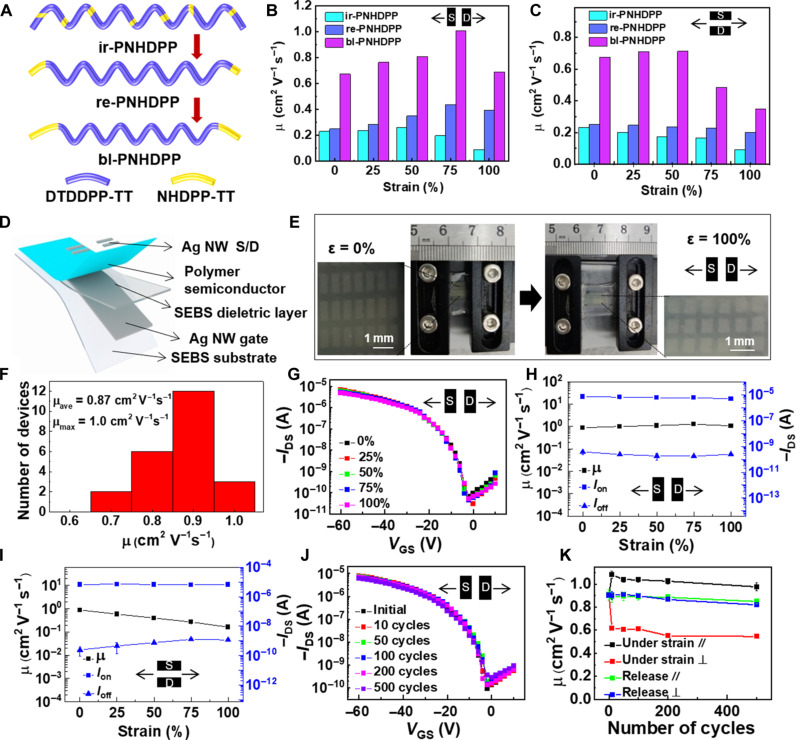
Optimization of polymer structures and fully stretchable transistors based on the optimized polymer bl-PNHDPP. (**A**) Schematic diagrams for polymer structure optimization. (**B**) Mobility comparison under various strains along the strain direction. (**C**) Mobility comparison under various strains along the perpendicular to strain direction. (**D**) Device structure of a BGTC stretchable transistor with the bl-PNHDPP semiconductor. (**E**) Images of the fully stretchable device under 0 and 100% strain. (**F**) Distributions of field-effect mobilities based on 23 devices. (**G**) Transfer curves of the stretchable transistor at different strains along the charge transport direction. (**H**) Field-effect mobility, on-current, and off-current as a function of various strains along the strain direction. (**I**) Field-effect mobility, on-current, and off-current as a function of various strains along the perpendicular to strain direction. (**J**) Transfer curves of the stretchable transistor under 25% strain after multiple stretching-releasing cycles along the charge transport direction. (**K**) Field-effect mobility as a function of stretching-releasing cycles along the strain direction and perpendicular to strain direction.

On the basis of the analysis and comparison of the regular and irregular polymers, we conjectured that the introduction of more continuous hydrogen bonds in the polymer skeleton were probably conducive to the improvement of electrical and mechanical properties. Therefore, we extended the chain length of the EHCDPP-TT fragment by prepolymerization and then obtained the block polymer bl-PEHCDP (Fig. 5A, figs. S46 to S48, and table S8), which can be thermally converted to bl-PNHDPP with a more regular and continuous hydrogen bond structure. The bl-PNHDPP polymer film exhibited a more porous morphology consisting of much bigger coiled nanofibers with substantial crystalline domains and continuous hydrogen bonds as revealed by AFM and cryo-TEM measurements (figs. S49 and S50), which held great potential to realize excellent balance between mobility and stretchability. The bl-PNHDPP polymer showed an average mobility of 0.68 cm^2^ V^−1^ s^−1^ and a maximum mobility of 0.77 cm^2^ V^−1^ s^−1^ extracted from 20 devices (fig. S51). Charge transport properties of the polymer films under strain were subsequently assessed. The bl-PNHDPP polymer exhibited remarkably improved mobilities parallel and perpendicular to the stretching direction compared with re-PNHDPP and ir-PNHDPP (Fig. 5, B and C). The mobility reached a maximum value (1.01 cm^2^ V^−1^ s^−1^) under 75% strain with about 1.5-fold increase relative to that of 0% strain in the parallel direction (Fig. 5B and fig. S52, A and E). The films maintained constant mobility under 50% strain and began to decrease with increasing strain along the perpendicular stretching direction (Fig. 5C and fig. S52, B and E). Furthermore, the polymer films also exhibited good mechanical reversibility (fig. S52, C to F). The mobility initially increased at 25% strain after 10 stretching-releasing cycles and then remained stable with the increase in the number of cycles along both the parallel and vertical stretching direction.

Last, fully stretchable transistors were fabricated based on bl-PNHDPP polymer films (Fig. 5, D and E). The stretchable transistor had the BGTC configuration with polystyrene-*block*-poly(ethylene-*ran*butylene)-*block*-polystyrene (SEBS 1062) as a substrate, silver nanowires (Ag NWs) as gate and source/drain electrodes, and SEBS (1052) as a dielectric layer. The detailed device fabrication procedures are described in Materials and Methods. Representative transfer curve and output curves of stretchable transistors suggested stable and ideal p-channel transistor characteristics (fig. S53). The devices had an average mobility of 0.87 cm^2^ V^−1^ s^−1^ and a maximum mobility of 1.01 cm^2^ V^−1^ s^−1^ at 0% strain (Fig. 5F). When the device was stretched along the charge transport direction, the field-effect mobility showed a pronounced increase (1.27 cm^2^ V^−1^ s^−1^) at 75% strain and a negligible decrease (1.08 cm^2^ V^−1^ s^−1^) at 100% strain (Fig. 5, G and H, fig. S54, and table S9), which are the unprecedented values for intrinsically stretchable and healable semiconducting polymers (*14*, *15*). In addition, the device showed excellent retention of mobilities in the perpendicular stretching direction (Fig. 5I, figs. S54 and 55A, and table S9). We further performed the stretching durability test for the bl-PNHDPP film at 25% strain. We observed that the field-effect mobility of our stretchable transistors did not suffer any noticeable degradation in performance even after 500 cycles in both parallel and perpendicular directions (Fig. 5, J and K, figs. S55B and S56, and table S10). Compared with other intrinsically stretchable polymer semiconductors reported so far, it showed one of the highest mobility values under 100% strain along both the parallel and perpendicular stretching direction (fig. S57) (*14*, *21*, *28*, *29*, *39*, *48*–*50*).

## DISCUSSION

In conclusion, we have developed intrinsically stretchable, healable, and high-mobility polymer semiconductors by in situ introducing continuous hydrogen bonding sites in the conjugated backbone through thermal conversion of polymer precursors, which were synthesized by stepwise polymerization. The designed polymer semiconductors neither break the conjugation of the polymer backbone nor introduce bulky softer side chains, thus retaining high charge carrier mobility with increasing stretchability and healability. The regular sequence structure, consisting of continuous *N*-alkyl segments and continuous N–H segments, facilitates the formation of loose, porous, and fibrous microstructures with a high degree of aggregation, long-range ordering, and increased chain dynamics, improving charge transport and stretchability simultaneously. On the other hand, the consecutive hydrogen bondings are able to undergo energy dissipation or healing mechanism through breakage or reconstruction of noncovalent bonds. Therefore, the re-PNHDPP polymer exhibited 1.8-fold enhanced carrier mobility under 75% strain, which is rare for intrinsically stretchable polymer semiconductors that usually have decreased mobilities when strain is applied. The damaged re-PNHDPP thin film can be almost recovered in terms of morphology and electrical characteristics after a solvent and thermal healing treatment. Furthermore, the block polymer bl-PNHDPP exhibited higher carrier mobilities at a strain resulting from the more regular sequence structure. The fully stretchable transistors based on bl-PNHDPP showed an excellent mobility up to 1.08 cm^2^ V^−1^ s^−1^ under 100% strain, which is the unprecedented value for intrinsically stretchable and healable semiconducting polymers. In addition, the stretchable transistors did not suffer from performance degradation even after being subjected to repeated strain. The incorporation of continuous hydrogen bonding sites in the conjugated backbone provides a promising strategy to solve the long-standing issue among charge transport, stretchability, and self-healing ability.

## MATERIALS AND METHODS

Chemicals and reagents were purchased from commercial sources and without further purification when used. For example, the ultradry chlorobenzene and *N*,*N*-dimethylformamide were purchased from Beijing Innochem Science & Technology Co. Ltd. and used as received. The TT monomer was purchased from TCI (Shanghai) Chemical Industrial Development Co. Ltd. DPP monomers DTDDPP and EHCDPP were successfully synthesized from 3,6-di(2-thienyl)2,5-dihydropyrrolo[3,4-*c*]pyrrole-1,4-dione via *N*-alkylation or *N*-carboxylation, respectively, and were followed by the bromination with *N*-bromosuccinimide (*22*, *37*, *51*). Polydimethylsiloxane (PDMS) precursors (Sylgard 184) were purchased from Dow Corning. SEBS (1221 and 1052) was from Asahi Kasei. The Ag NW solution (average diameter, 25 to 30 nm; length, 10 to 30 μm) was from Zhejiang Kechuang Advanced Materials Technology Co. Ltd. All processing solvents were purchased from Aladdin. All of the above materials were used without further purification for substrate and device fabrication.

### Synthesis of various conjugated polymers

All of the studied conjugated polymers based on DPP main-chain structures were synthesized by the Stille coupling polycondensation with dibrominated DPP monomers and stannylated compounds (TT) by control of feeding sequences of relative monomers (*51*). Details of all synthetic procedures are available in the Supplementary Materials (figs. S1, S2, and S46; molecular weight and dispersity of the polymers are given in table S1, and element analysis results are shown in tables S2 and S8).

### Thin film preparation

Wafers with 300-nm silicon dioxide on n^++^ Si were cleaned with deionized water, acetone, and isopropanol, in that order, and then modified with an OTS self-assembled monolayer according to a previously reported method (*13*). Polymers were dissolved in chlorobenzene (7 mg/ml) at 80°C, and then the polymer solutions were spin coated on OTS-treated SiO_2_ wafers at 1500 rpm for 60 s. The thickness of the polymer films was controlled at about 30 nm. The obtained semiconducting films were annealed at 200°C in nitrogen for 30 min to thermally remove the EHC group.

### Material characterization

PDMS films were prepared at a ratio of 15:1 (base:cross-linker, w/w) and cured overnight at 80°C and then cut into a slab (1 by 2 cm) used for the laminating substrate to transfer the polymer thin films. The polymer thin films peeled from OTS-treated SiO_2_ wafers with the PDMS slab were transferred onto SiO_2_/Si substrates. Then, the gold source and drain electrodes (40 nm in thickness) were deposited with shadow masks (*W*/*L* = 8.2). All samples have the BGTC device architecture. Electrical characterization of the OFETs was conducted with Keithley 4200 in a N_2_ glove box. The dielectric capacitance of the SiO_2_ gate was 10 nF cm^−2^.

UV-vis/NIR absorption spectra were measured with UV-vis/NIR spectroscopy in the range of 300 to 1100 nm (Agilent Cary 6000i UV-Vis-NIR spectrometer). For dichroic ratio measurement, the PDMS slab was chosen to peel off the polymer films from OTS-treated SiO_2_ substrates and then stretched to certain strains. A polarizer was used to obtain the polarized light parallel and perpendicular to the strain direction.

GIWAXS data were obtained at the 1W1A Diffuse X-ray Scattering Station, Beijing Synchrotron Radiation Facility (BSRF-1W1A). All the thin films prepared onto Si/SiO_2_ substrates were irradiated at a fixed angle of 0.16° and exposed for 60 s. The rDoC was derived from (200) peaks of the thin film.

### Self-healing process

The films were damaged to form nanocracks with a strain of 50% for ir-PNHDPP and 100% for re-PNHDPP up to 10 cycles. Then, the sample initially treated with CHCl_3_ was vapor annealed for 10 min and then thermally annealed in a N_2_ glove box at 120°C for 10 min. This process was repeated three times.

### Fully stretchable transistors

SEBS H1221 (from Asahi Kasei) was dissolved in toluene (250 mg ml^−1^) at 80°C and then poured onto Si/SiO_2_ substrates (2 by 2 cm), and then the obtained films were peeled and used as substrates for fully stretchable transistors. The Ag NW solution diluted to 0.2 mg ml^−1^ with deionized water was casted onto the Si/SiO_2_ wafers patterned with Kapton tape. The Ag NW gate electrodes were subsequently transferred onto SEBS substrates. SEBS H1052 (from Asahi Kasei) was dissolved in toluene (70 mg ml^−1^) and filtered with a 0.22-μm filter. The solution was spin coated onto the OTS-Si/SiO_2_ substrates at 1000 rpm for 60 s. The resulting film was annealed in a vacuum drying oven at 100°C for 1 hour. This SEBS dielectric layer was then transferred onto the Ag NW gate electrode. Next, the semiconducting polymer film was then transferred onto the dielectric layer. Last, the diluted Ag NW solution was then spray coated onto the semiconducting layer through a shadow mask with a channel length (*L*) of 1000 μm and a width (*W*) of 200 μm. The mobilities were calculated with measured device geometry and dielectric capacitance under strain (table S11).
