# Supplementary material for: In situ continuous hydrogen-bonded engineering for intrinsically stretchable and healable high-mobility polymer semiconductors
Source: Sci Adv. 2024 Oct 2;10(40):eadq0171. doi: 10.1126/sciadv.adq0171 (PMC11446264; doi:10.1126/sciadv.adq0171)
Supplement: Supplementary file 1 — Supplementary Text Figs. S1 to S57 Tables S1 to S11 [file sciadv.adq0171_sm.pdf]

Science Advances
AAAS

Supplementary Materials for

In situ continuous hydrogen-bonded engineering for intrinsically stretchable and healable high-mobility polymer semiconductors

Haoguo Yue *et al.*

Corresponding author: Yonggang Zhen, zhenyg@buct.edu.cn

Sci. Adv. **10**, eadq0171 (2024)
DOI: 10.1126/sciadv.adq0171

This PDF file includes:

Supplementary Text
Figs. S1 to S57
Tables S1 to S11

1. Instruments and methods

Elemental analyses (EA) were performed on Carlo-Erba-1106 instrument. Gel permeation chromatography (GPC) was performed on Agilent PL-GPC 220 instrument using 1,2,4-trichlorobenzene as an eluent (1.0 mL/min) at 160°C, polystyrene was utilized as the calibration standard. The solid-state ^{1}H NMR spectrum of the polymers were characterized by Bruker NEO 600 WB instrument (600 MHz, spinning frequency of 60 kHz, ultra-high speed magic angle rotating 1.3 mm probe) for proving the continuous hydrogen bonding sites. The variable temperature ^{1}H NMR spectra of polymers were performed in 1,1,2,2-tetrachloroethane-d2 at 120°C by the Bruker AV Ш 500WB instrument. UV-vis-NIR absorption spectra of films were collected on the Agilent Cary 6000i UV-Vis/NIR spectrometer, all thin films were spin-coated from chlorobenzene solution with a concentration of 5.0 mg/mL on quartz plates. Atomic Force Microscope (AFM) images were taken with Bruker DMFASTSCAN2-SYS at tapping mode. Optical microscope images were recorded using an Olympus-BX53M microscope. Ac chip calorimetry was used to detect and quantify the glass transition in different samples (*46*). The XI-392 sensor from Xensor Integration with a heated area of 100 mm × 100 mm was used. A heating rate of 1 K/min and a frequency of 10 Hz was used to probe the glass transition behavior of samples. All measurements were performed under a dry nitrogen atmosphere at ambient pressure. The FET device performances were evaluated on a Keithley 4200 SCS semiconductor S3 parameter analyzer. All measuring processes were performed under ambient or nitrogen conditions.

2. Materials synthesis and characterization

Synthetic route:

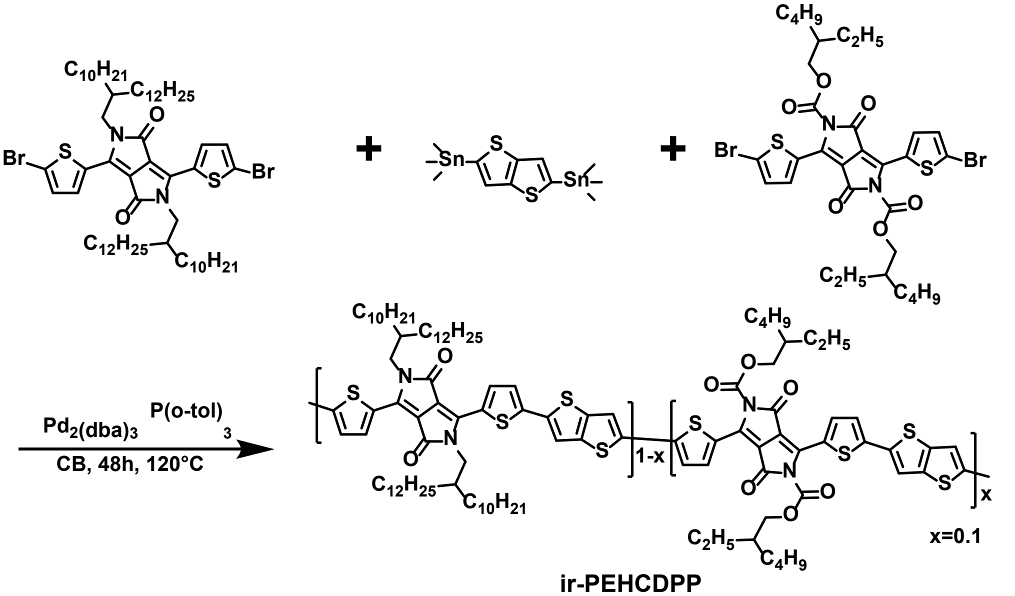

Fig. S1. Synthetic route of irregular polymer ir-PEHCDPP.

Synthesis of irregular polymer ir-PEHCDPP: 2,5-bis(trimethylstannyl)thieno[3,2-b]thiophene (TT, 93.2 mg, 0.2 mmol), 3,6-bis-(5-bromo-thiophen-2-yl)-2,5-bis(2-decyltetradecyl)-1,4-dioxo-pyrrolo[3,4-c]pyrrole (DPP, 203.6 mg, 0.18 mmol), 3,6-bis(5-bromo-thiophen-2-yl)-2,5-bis(2-ethylhexyl-carboxylate)-1,4-dioxo-pyrrolo[3,4-c]pyrrole (EHCDPP, 15.6 g, 0.02 mmol), $Pd_2(dba)_3$ (6 mg), $P(o\text{-}tol)_3$ (16.4 mg), and dry chlorobenzene (4 mL) were added to a 50 mL fused Schlenk tube, which was charged with nitrogen through a freeze-pump-thaw cycle for

three times. Then, the reaction mixture was stirred for 48 h at 120°C under nitrogen atmosphere. Next, the reaction mixture was cooled down to room temperature, poured into a beaker containing 120 mL methanol and 3 mL hydrochloric acid, and then stirred for 3 h. The crude polymer was precipitated in methanol, collected by filtration, then loaded to an extraction thimble. The thimble was left into a Soxhlet extractor, and the crude polymer was successively washed with methanol, acetone and hexane. The resulting polymer was collected by trichloromethane, concentrated and then precipitated into methanol. The precipitated polymer was collected by filtration and dried under high vacuum overnight, yielding the target polymer as dark green solid (162 mg, 75%). GPC: M_n = 60.6 kDa, M_w = 217.2 kDa, PDI = 3.59.

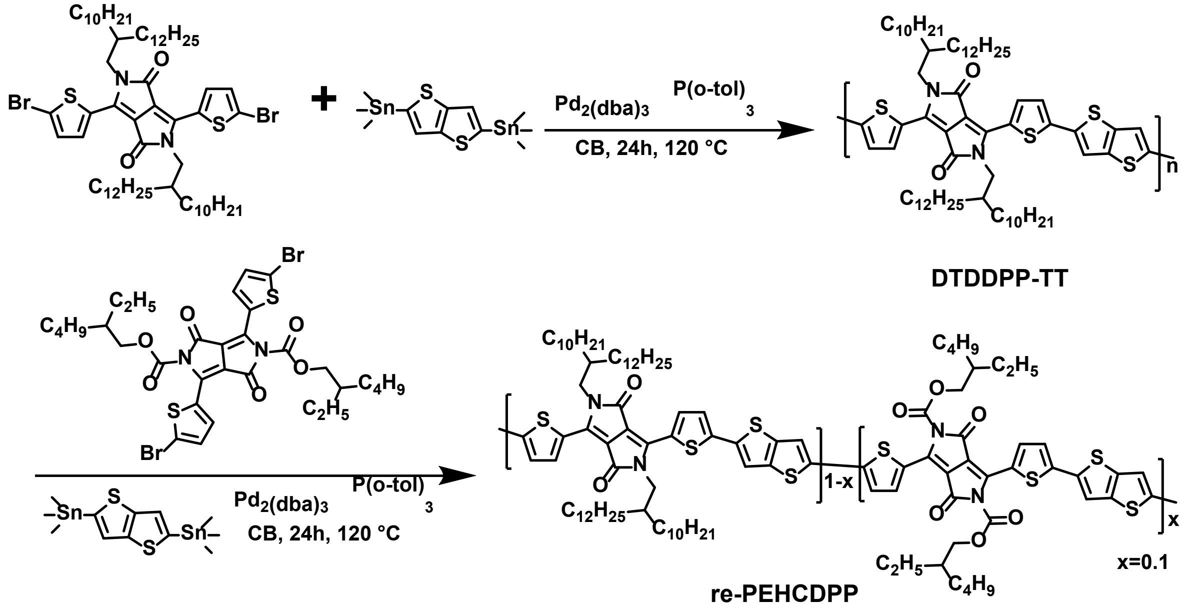

Fig. S2. Synthetic route of regular polymers re-PEHCDPP.

Synthesis of regular polymer re-PEHCDPP: The synthesis procedure of regular polymer PEHCTT is different from that of irregular polymer PEHCTT. The whole synthesis process is mainly divided into two steps for irregular polymer PEHCTT. Firstly, 2,5-bis(trimethylstannyl)thieno[3,2-b]thiophene (TT, 83.9 mg, 0.18 mmol), 3,6-bis-(5-bromo-thiophen-2-yl)-2,5-bis(2-decyltetradecyl)-1,4-dioxo-pyrrolo[3,4-c]pyrrole (DPP, 203.6 mg, 0.18 mmol), $Pd_2(dba)_3$ (5.4 mg), $P(o\text{-}tol)_3$ (14.7 mg), and dry chlorobenzene (3.5 mL) were added to a 50 mL fused Schlenk tube, which was charged with nitrogen through a freeze-pump-thaw cycle for three times. The reaction mixture was stirred for 24 h at 120°C under nitrogen atmosphere. Secondly, 2,5-bis(trimethylstannyl)thieno[3,2-b]thiophene (TT, 9.3 mg, 0.02 mmol), 3,6-bis(5-bromo-thiophen-2-yl)-2,5-bis(2-ethylhexyl-carboxylate)-1,4-dioxo-pyrrolo[3,4-c]pyrrole (EHC-DPP, 15.6 g, 0.02 mmol), $Pd_2(dba)_3$ (0.6 mg), $P(o\text{-}tol)_3$ (1.6 mg), and dry chlorobenzene (1.5 mL) were added to a 10 mL fused Schlenk tube, which was charged with nitrogen through a freeze-pump-thaw cycle for three times, quickly transferred to the first reaction system (50mL reaction bottle), and then stirred for 24 h at 120°C under nitrogen atmosphere. Next, the post-processing procedure was similar to that of ir-PEHCDPP and finally obtained a glossy film after removing the solvent (175 mg, 81%). GPC: M_n = 49.5 kDa, M_w= 126.8 kDa, PDI = 2.56.

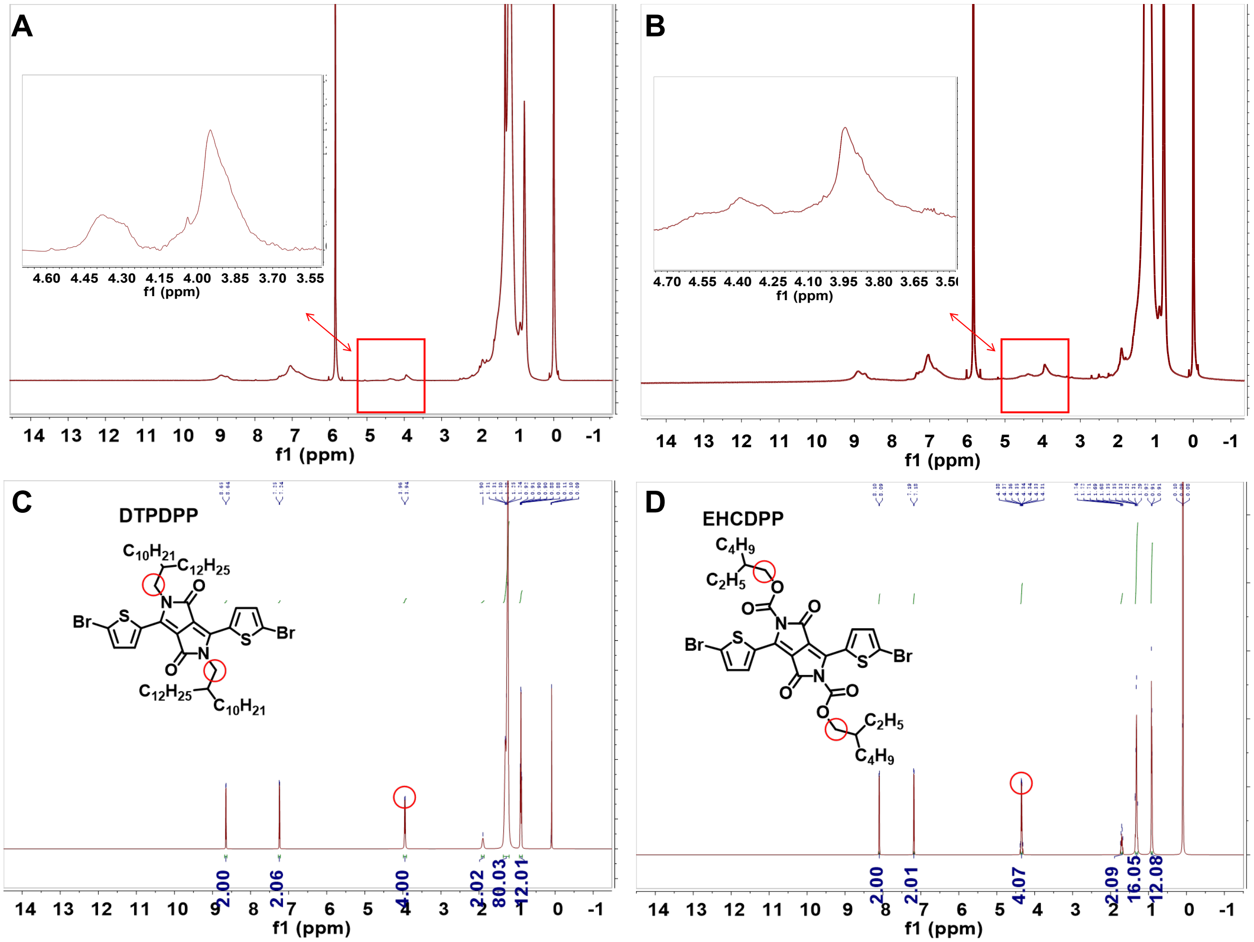

Fig. S3. **^{1}H NMR characterization of polymers and monomers.** The high-temperature ^{1}H NMR spectroscopy of re-PEHCDPP (**A**) and ir-PEHCDPP (**B**). The ^{1}H NMR spectroscopy of the related monomers of DTPDPP (**C**) and EHCDPP (**D**).

Table S1. Molecular weight characterization of polymers re-PEHCDPP and ir-PEHCDPP [a]

Polymer	M_n[b)] [kDa]	M_w[kDa]	PDI
re-PEHCDPP	49.5	126.8	2.56
ir-PEHCDPP	60.6	217.2	3.59

a)Abbreviations: M_n, number-average molecular weight; M_w, weight-average molecular weight. PDI, polymer dispersity index. b)Determined by high-temperature gel permeation chromatography (GPC) using 1,2,4-trichlorobenzene as the eluent at 160°C and calibrated using polystyrene standards.

Table S2. Elemental analysis of polymers before and after thermal annealing

Composite	C	H	N	S	Average ratio of EHC or NH group[a]	Data sources
PEHCDPP	72.09%	9.18%	2.66%	12.15%	10.00%	Theoretical[b]
PNHDPP	71.59%	8.88%	2.91%	13.30%	10.00%	Theoretical[c]
re-PEHCDPP	72.14%	9.21%	2.65%	12.11%	9.46%	Measured[d]
re-PNHDPP	71.68%	8.92%	2.89%	13.18%	9.42%	Measured[d]
ir-PEHCDPP	71.97%	9.15%	2.66%	12.19%	10.64%	Measured[d]
ir-PNHDPP	71.48%	8.84%	2.93%	13.42%	10.59%	Measured[d]

[a)] Derived from the measured C, H, N, S element contents and calculated based on the theoretical ratio respectively. [b)] Derived from 10% part NHDPP-TT and 90% part DTDDPP-TT. [c)] Derived from 10% part NHDPP-TT and 90% part DTDDPP-TT. [d)] Determined by element analyzer.

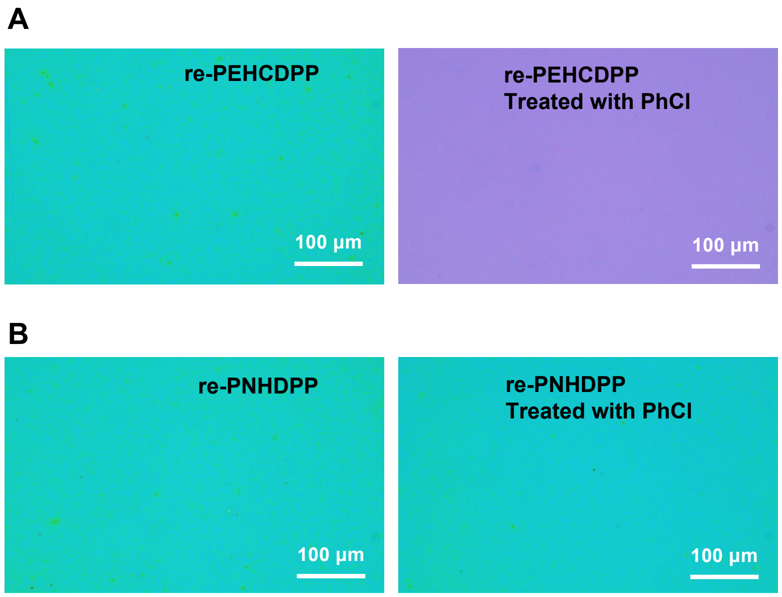

Fig. S4. Solvent resistance for regular polymer films characterized by optical microscope. Optical microscopic images of (**A**) re-PEHCDPP and (**B**) re-PNHDPP films before and after treatment with chlorobenzene solvent. (Scale bar: 100 μm) The unannealed film was washed off by the solvent, but the re-PNHDPP film was basically unchanged after solvent treatment.

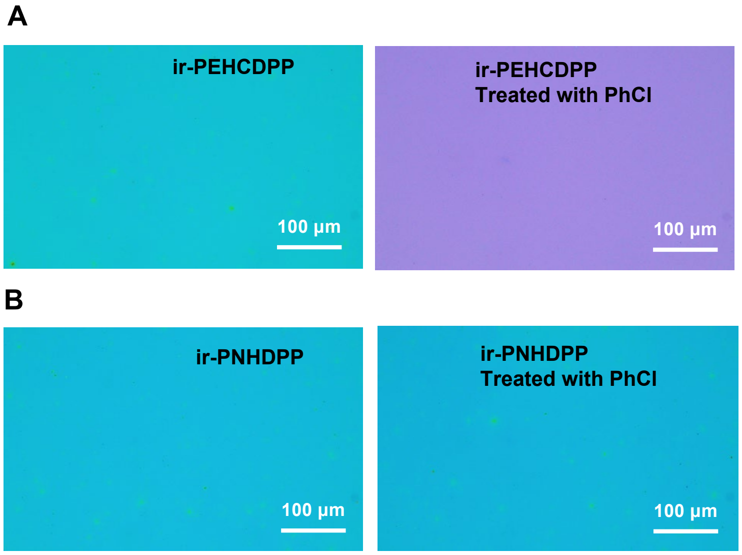

Fig. S5. **Solvent resistance for irregular polymer films characterized by optical microscope.** Optical microscopic images of (**A**) ir-PEHCDPP and (**B**) ir-PNHDPP films before and after treatment with chlorobenzene solvent. (Scale bar: 100 μm) The unannealed film was washed off by the solvent, but the ir-PNHDPP film was basically unchanged after solvent treatment.

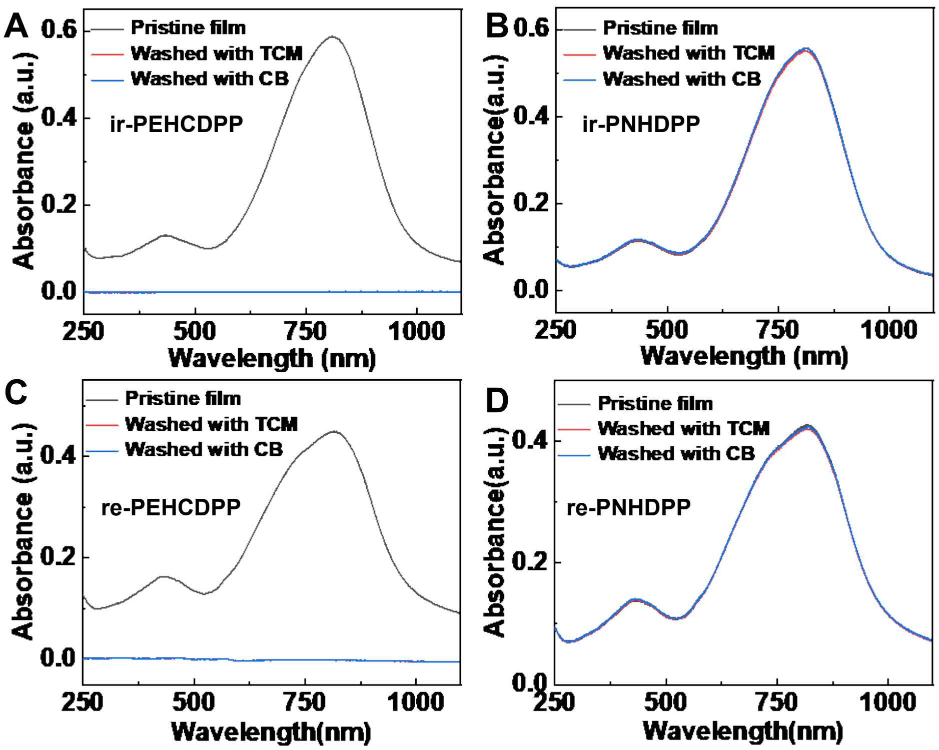

Fig. S6. **Solvent resistance measurement for polymer films characterized by UV-Vis spectrum.** The UV-Vis spectrum of polymer pristine films, and the films washed by trichloromethane (TCM) and chlorobenzene (CB) for ir-PEHCDPP (**A**), ir-PNHDPP (**B**), re-PEHCDPP (**C**) and re-PNHDPP (**D**).

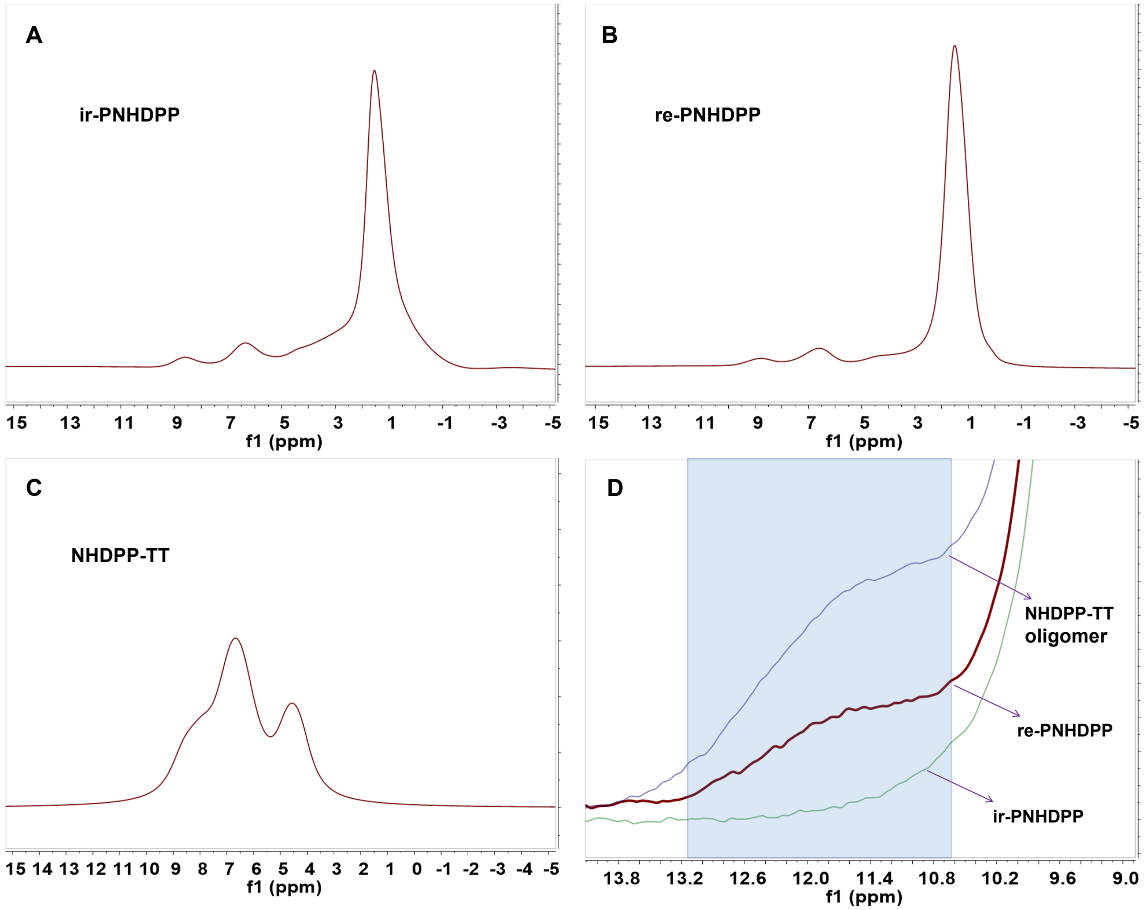

Fig. S7. Solid-state ^{1}H NMR characterization of polymers. Solid-state ^{1}H NMR spectrum of the ir-PNHDPP (**A**), re-PNHDPP (**B**) reference NHDPP-TT oligomer (**C**) (600 MHz, spinning frequency of 60 kHz, ultra-high speed magic angle rotating 1.3 mm probe), and partial enlarged image (**D**) of three polymers for comparing the characteristic peak of continuous hydrogen bonding sites.

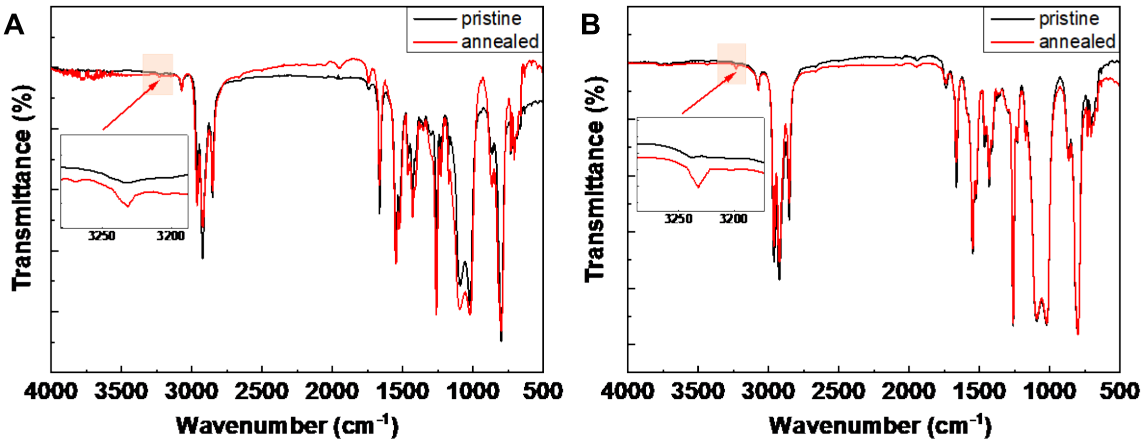

Fig. S8. The formation of NH- bonds confirmed by FT-IR. FT-IR spectra for the pristine films and after thermal treatment for polymers re-PEHCDPP (**A**) and ir-PEHCDPP (**B**).

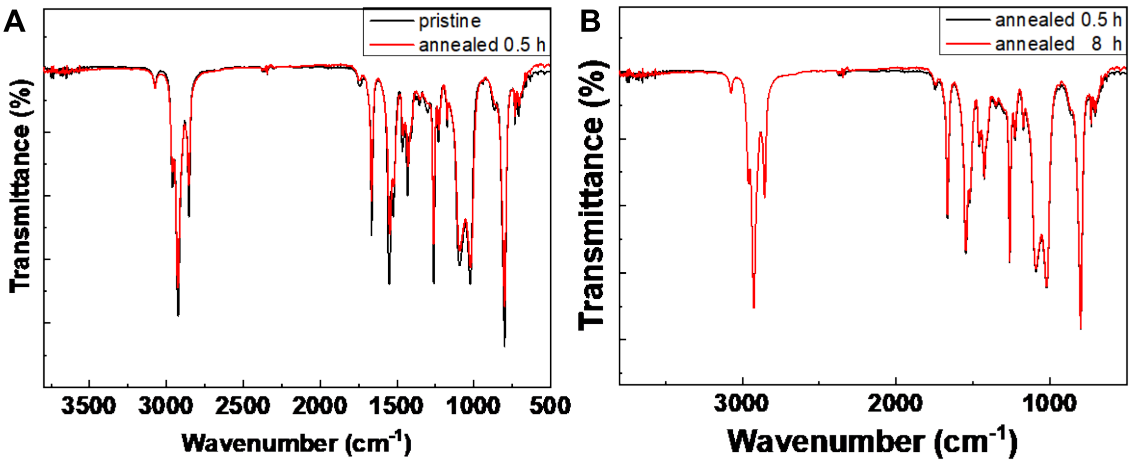

Fig. S9. **The in-situ FT-IR spectrum of regular polymer at different annealed time.** (**A**) In-situ FT-IR spectra of re-PEHCDPP for the pristine film and after thermal treatment (annealed 0.5 h). (**B**) In-situ FT-IR spectra of re-PNHDPP after thermal treatment 0.5 h and 8 h at 200°C.

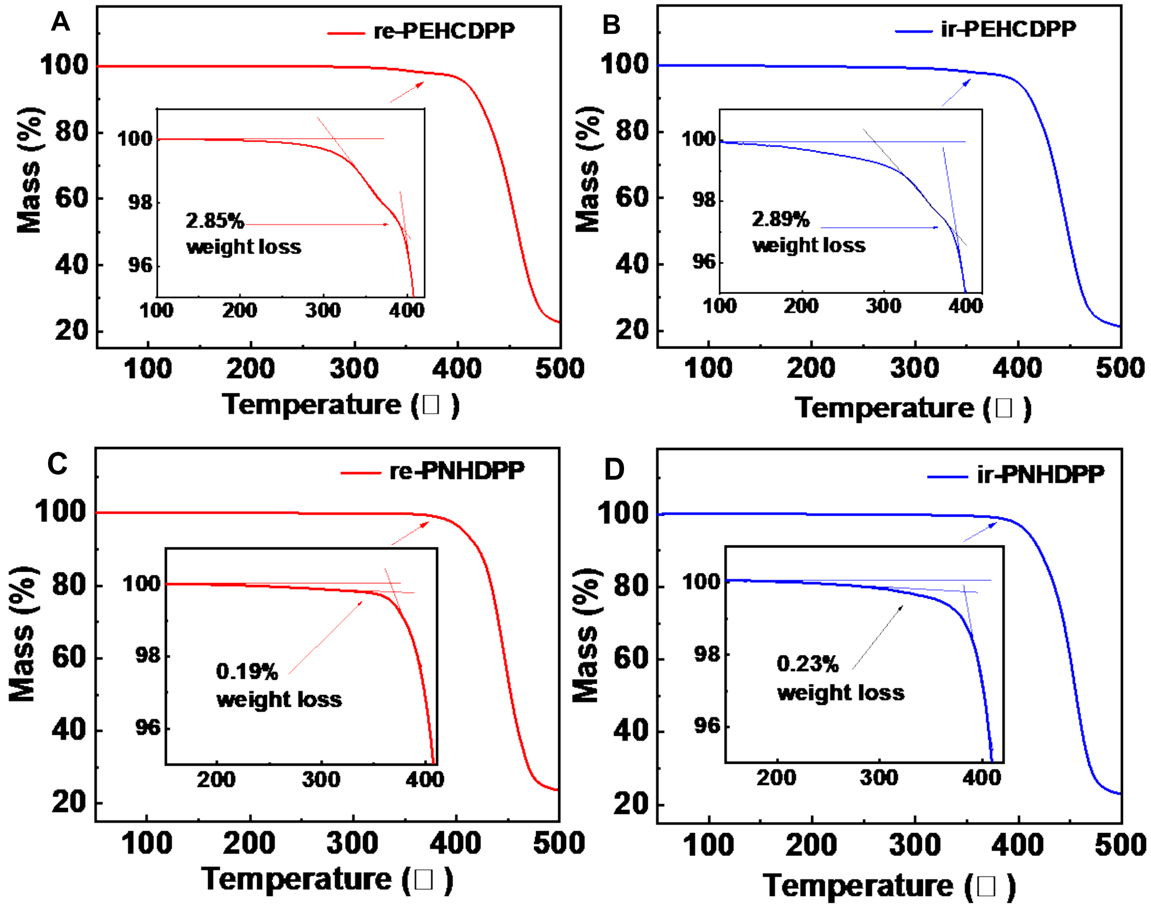

Fig. S10. **TGA characteristics of polymers**. (**A**) re-PEHCDPP. (**B**) ir-PEHCDPP. (**C**) re-PNHDPP. (**D**) ir-PNHDPP. The heating rate was set as 10°C min^{-1}.

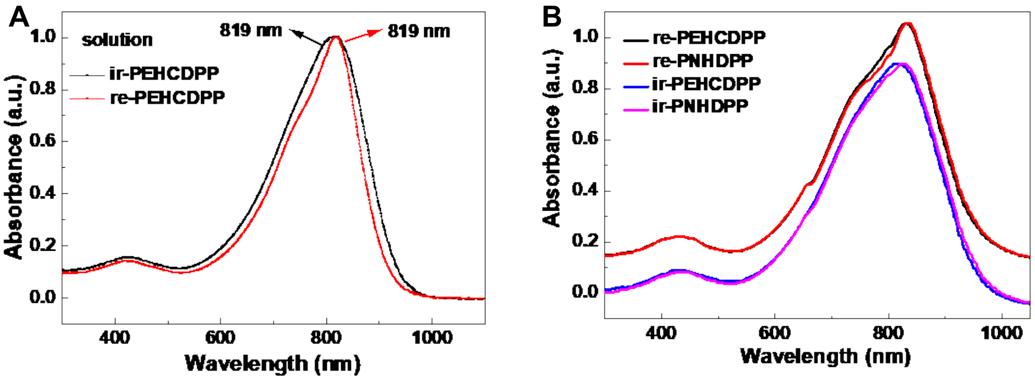

Fig. S11. **UV–vis/NIR absorption spectra of the polymers in solution and film.** (**A**) Normalized UV–vis/NIR absorption spectra of ir-PEHCDPP and re-PEHCDPP in chlorobenzene solution; (**B**) Normalized UV–vis/NIR absorption spectra of thin films of ir-PEHCDPP, re-PEHCDPP, ir-PNHDPP and re-PNHDPP.

3. Improved mechanical properties and self-healing ability without compromising mobility

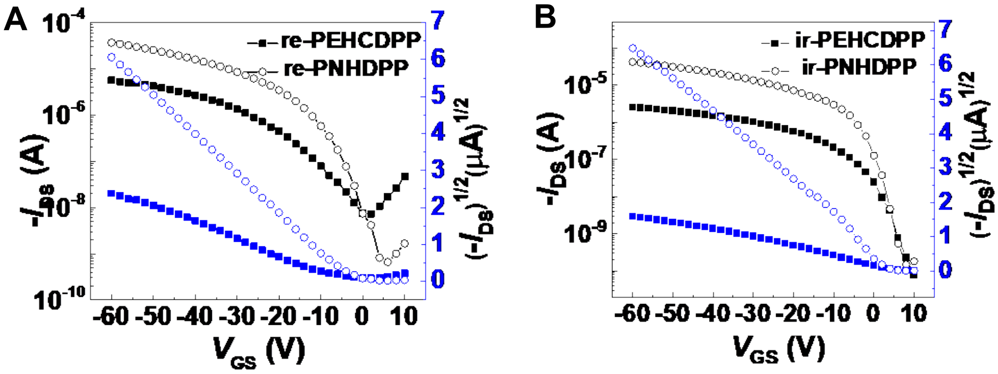

Fig. S12. Transfer curves of the polymer thin films in rigid transistor configuration. (**A**). re-PEHCDPP and re-PNHDPP. (**B**). ir-PEHCDPP and ir-PNHDPP.

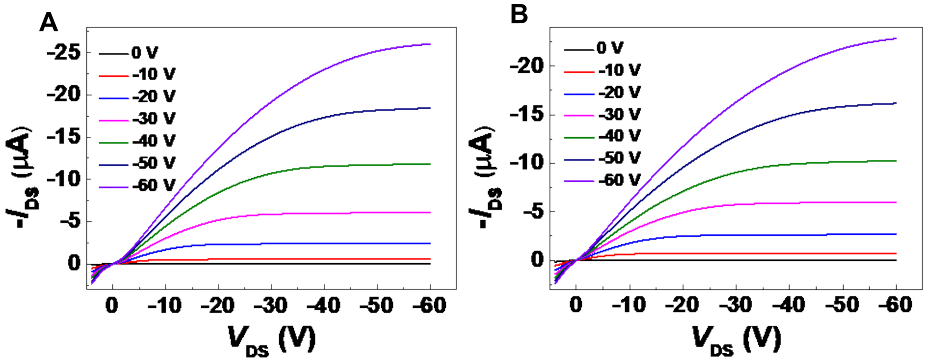

Fig. S13. **Output curves of the polymer thin films in rigid transistor configuration.** (**A**) re-PNHDPP film, (**B**) ir-PNHDPP film.

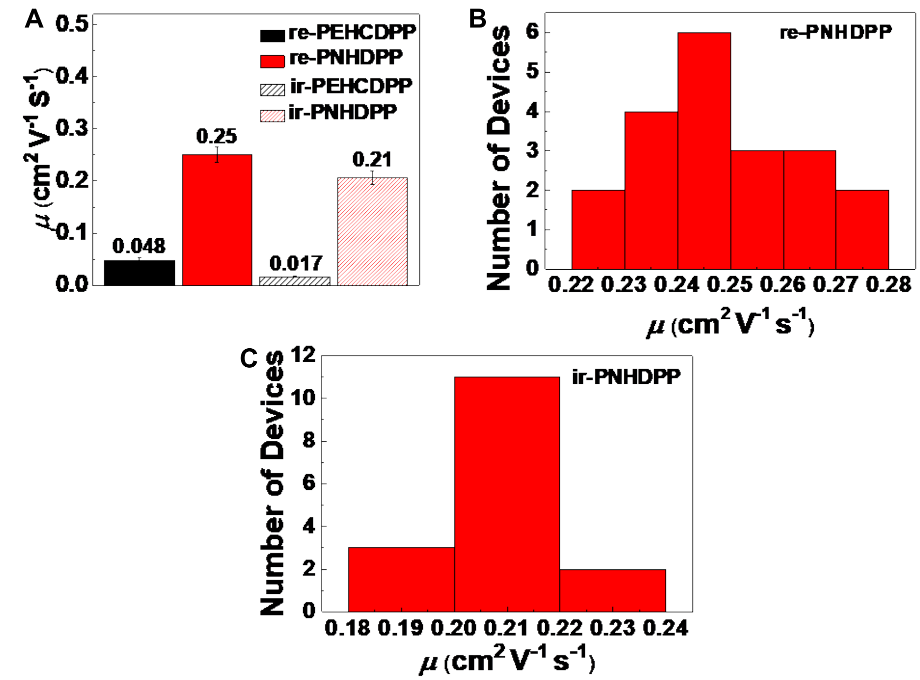

Fig. S14. Field-effect mobilities of the polymer films in rigid transistor configuration. Field-effect mobilities of the polymer films (**A**) and distribution of the field-effect mobility for re-PNHDPP film (**B**) and ir-PNHDPP film (**C**).

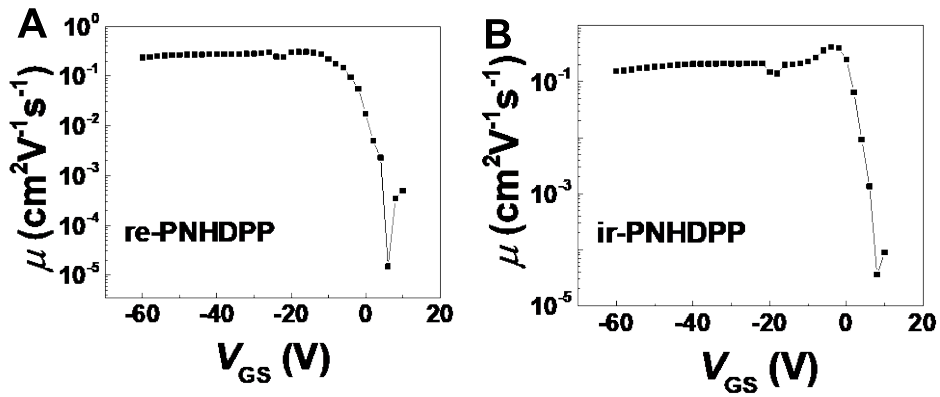

Fig. S15. Field-effect mobility as a function gate voltage of OFETs. (**A**) re-PNHDPP. (**B**) ir-PNHDPP.

Table S3. OFETs parameters of thin films of re-PNHDPP under different strains

Strain	Direction	μ_{ave} (cm² V⁻¹ s⁻¹)	V_{th} (V)	I_{on}/I_{off}	*SS* (V dec⁻¹)	*r* (%)
0%		0.250 ± 0.011	0.34 ± 2.15	10^4-10^5	3.15 ± 0.25	94.36 ± 1.84
25%	//	0.285 ± 0.021	-1.54 ± 2.01		3.82 ± 0.72	92.54 ± 1.99
	⊥	0.245± 0.008	-1.62 ± 0.97		3.80 ± 0.79	93.14 ± 2.81
50%	//	0.351 ± 0.01	-1.82 ± 0.52		3.89 ± 0.81	94.16 ± 1.67
	⊥	0.236 ± 0.003	-3.20 ± 0.92		4.07 ± 0.75	93.02 ± 1.76
75%	//	0.439 ± 0.012	-4.36 ± 0.65		5.07 ± 0.65	88.50 ± 1.18
	⊥	0.229 ± 0.013	-2.98 ± 0.29		4.26 ± 0.32	92.74 ± 1.01
100%	//	0.394 ± 0.01	-3.60± 0.92		5.67 ± 0.21	88.56 ± 1.64
	⊥	0.201 ± 0.015	-4.02 ± 0.57		4.66 ± 0.45	88.58 ± 0.63

Note: In order to gauge the reliability of the claimed mobility, we further calculated the reliability factor r according to previous report. It is defined as the ratio of the maximum channel conductivity experimentally achieved in a FET at the maximum gate voltage to the maximum channel conductivity expected in a correctly functioning ideal FET with the claimed carrier mobility. It can be expressed as:

$$r = \left(\frac{\sqrt{|I_{\mathrm{DS}}|^{\max}} - \sqrt{|I_{\mathrm{DS}}^{0}|}}{|V_{GS}|^{\max}}\right)^2 \Bigg/ \left(\frac{\partial\sqrt{|I_{DS}|}}{\partial V_{GS}}\right)^2$$

, where $|I_{\mathrm{DS}}|^{\max}$ is the experimental maximum source-drain current reached at the maximum gate voltage, I_{DS}^{0} denotes the source-drain current at $V_{\mathrm{GS}} = 0$ V.

Table S4. OFETs parameters of thin films of ir-PNHDPP under different strains.

Strain	Direction	μ_{ave} (cm² V⁻¹ s⁻¹)	V_{th} (V)	I_{on}/I_{off}	*SS* (V dec⁻¹)	*r* (%)
0%		0.206 ± 0.013	3.86 ± 1.29	10^5-10^6	2.30 ± 0.17	103.13 ± 2.59
25%	//	0.285 ± 0.021	0.52 ± 0.74		2.73 ± 0.19	100.06 ± 1.81
	⊥	0.245± 0.008	-0.86 ± 0.26		3.13 ± 0.26	101.55 ± 2.29
50%	//	0.351 ± 0.01	0.86 ± 2.33		3.45 ± 0.24	98.88 ± 2.56
	⊥	0.236 ± 0.003	-0.52 ± 1.12		3.23 ± 0.35	101.08 ± 0.81
75%	//	0.439 ± 0.012	-0.11 ± 0.65		3.10 ± 0.26	99.32 ± 1.13
	⊥	0.229 ± 0.013	-1.71 ± 0.61		3.44 ± 0.19	100.04 ± 1.14
100%	//	0.394 ± 0.01	-1.52± 0.71		3.07 ± 0.14	103.13 ± 0.46
	⊥	0.201 ± 0.015	-0.28 ± 0.98		3.02 ± 0.08	102.48 ± 1.18

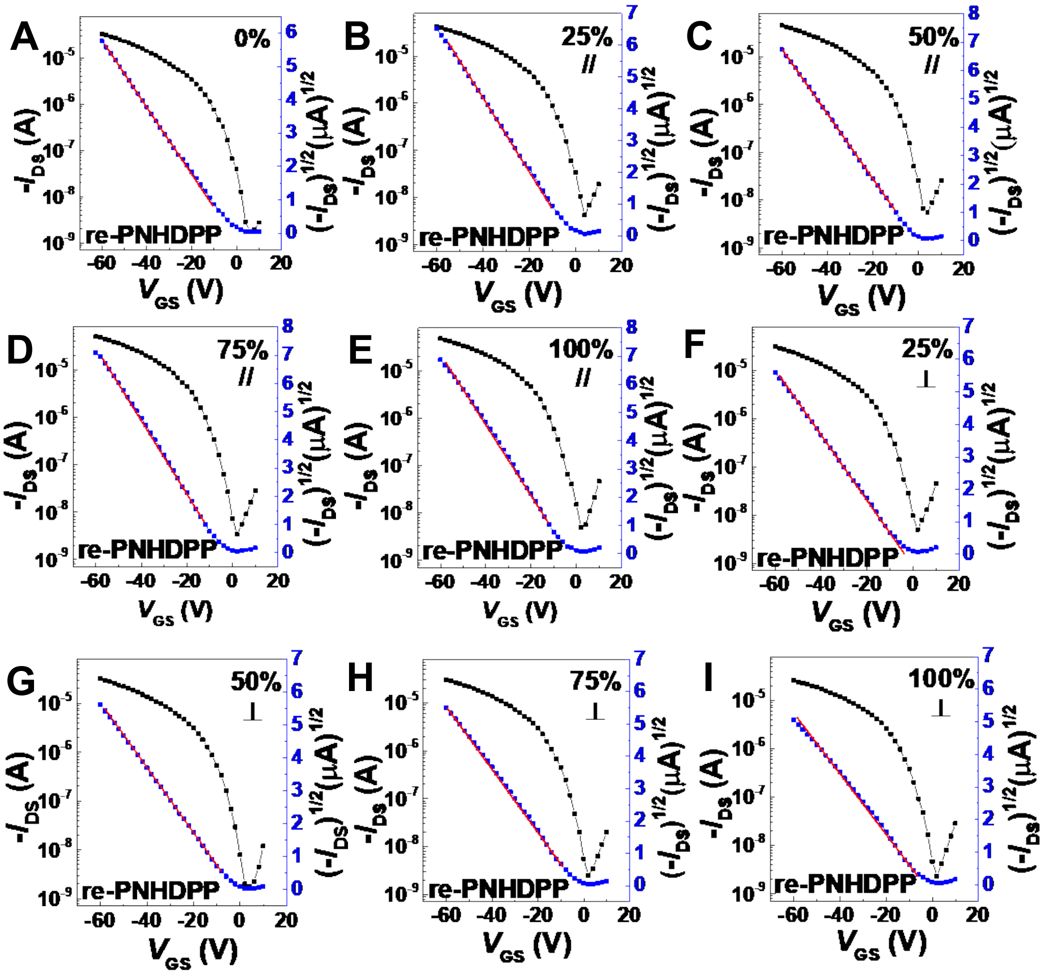

Fig. S16. Representative transfer curves of OFETs of re-PNHDPP under different strains and the respective plots of $(I_{DS})^{1/2}$ vs V_{GS}. Parallel represents charge transporting direction parallel to strain direction, and vertical represents charge transporting direction perpendicular to strain direction. The source-to-drain voltage is set as −60 V. The red line indicates the mobility extraction.

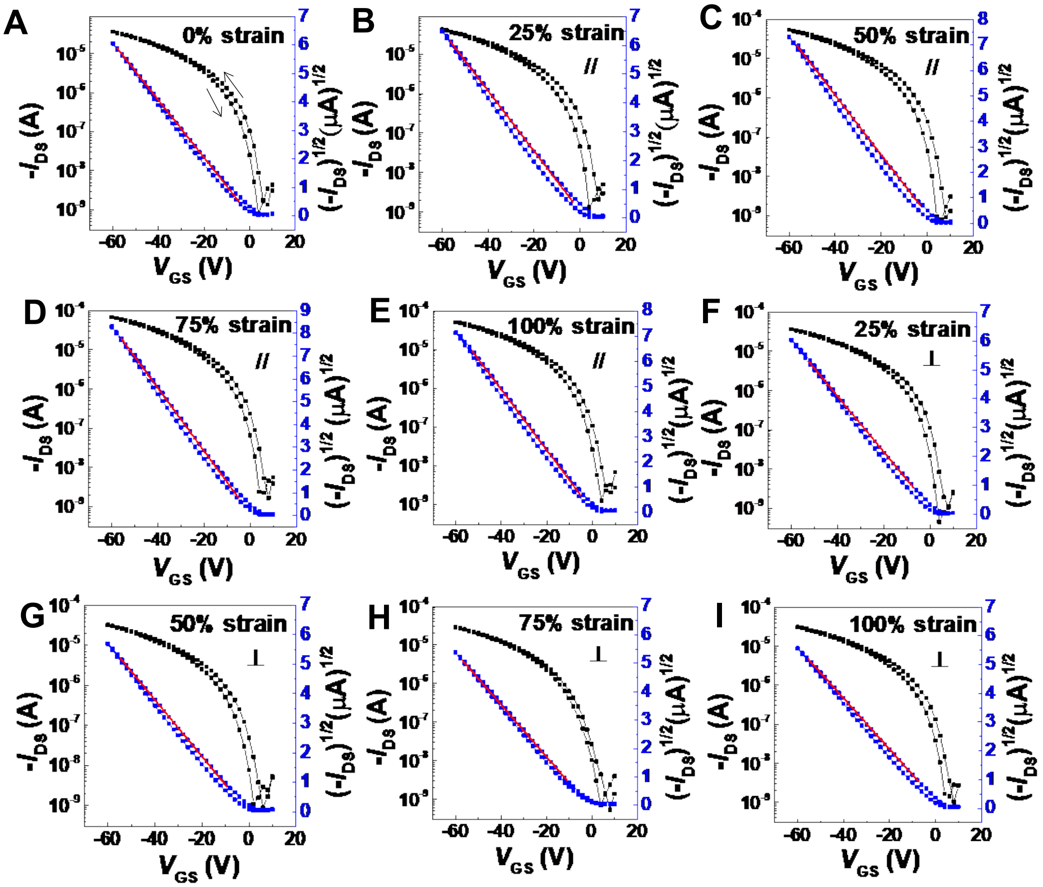

Fig. S17. Hysteresis curves (V_{DS} = −60 V) of the re-PNHDPP films under different strains.

.

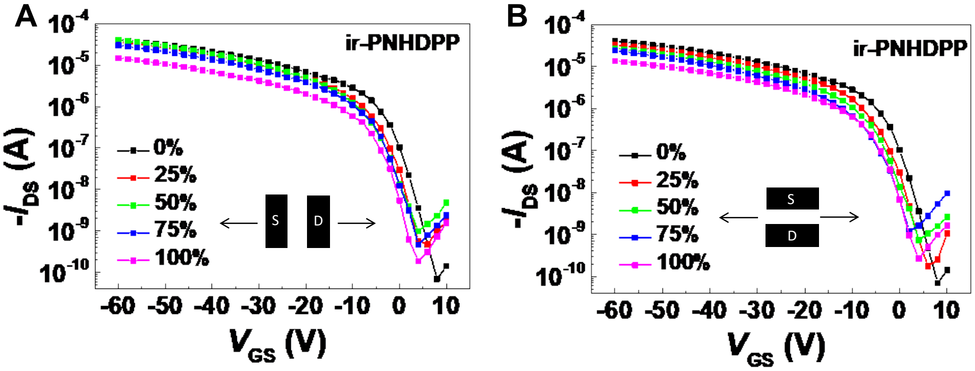

Fig. S18. Electrical characteristics of the polymer thin films at different strains in rigid transistor configuration. Transfer curves of ir-PNHDPP films at different strains along the charge transport direction (**A**) and perpendicular to charge transport direction (**B**).

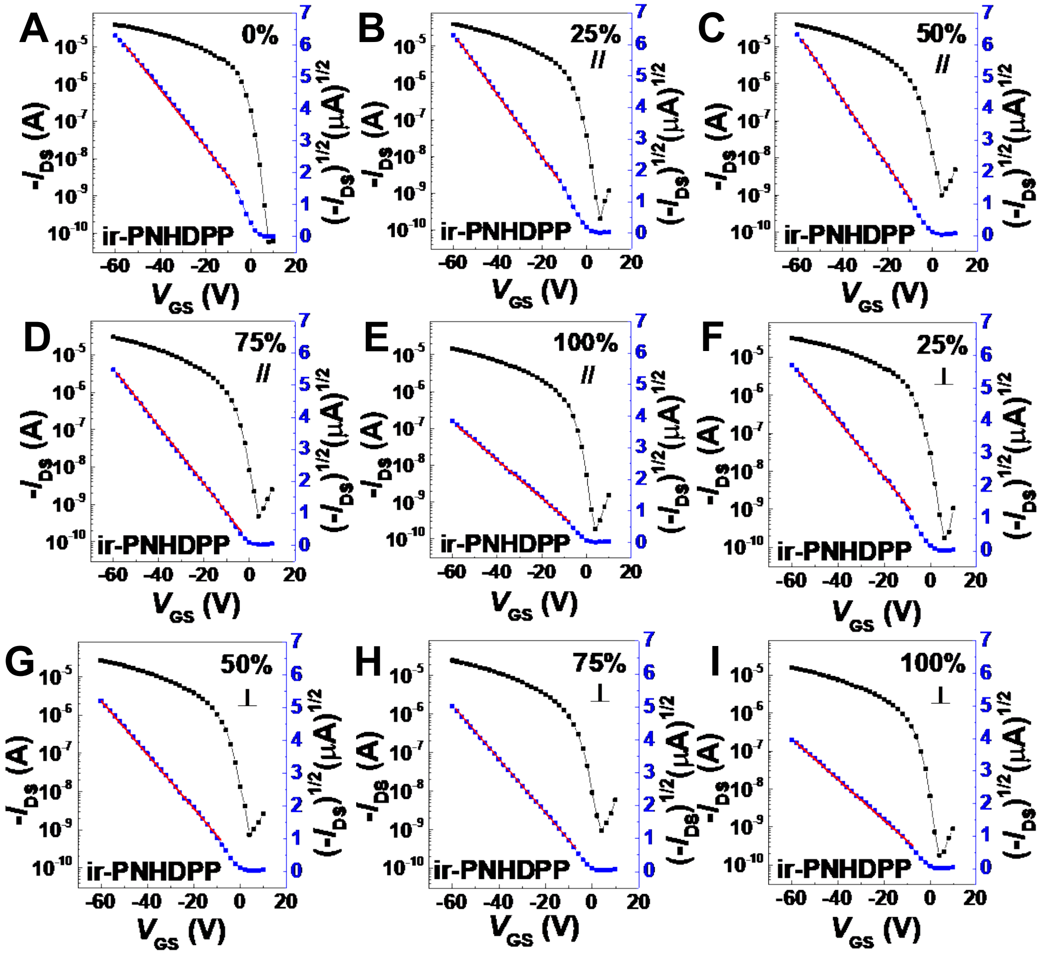

Fig. S19. Representative transfer curves of OFETs of ir-PNHDPP under different strains and the respective plots of (I_{DS})$^{1/2}$ vs V_{GS}. Parallel represents charge transporting direction parallel to strain direction, and vertical represents charge transporting direction perpendicular to strain direction. The source-to-drain voltage is set as −60 V. The red line indicates the mobility extraction.

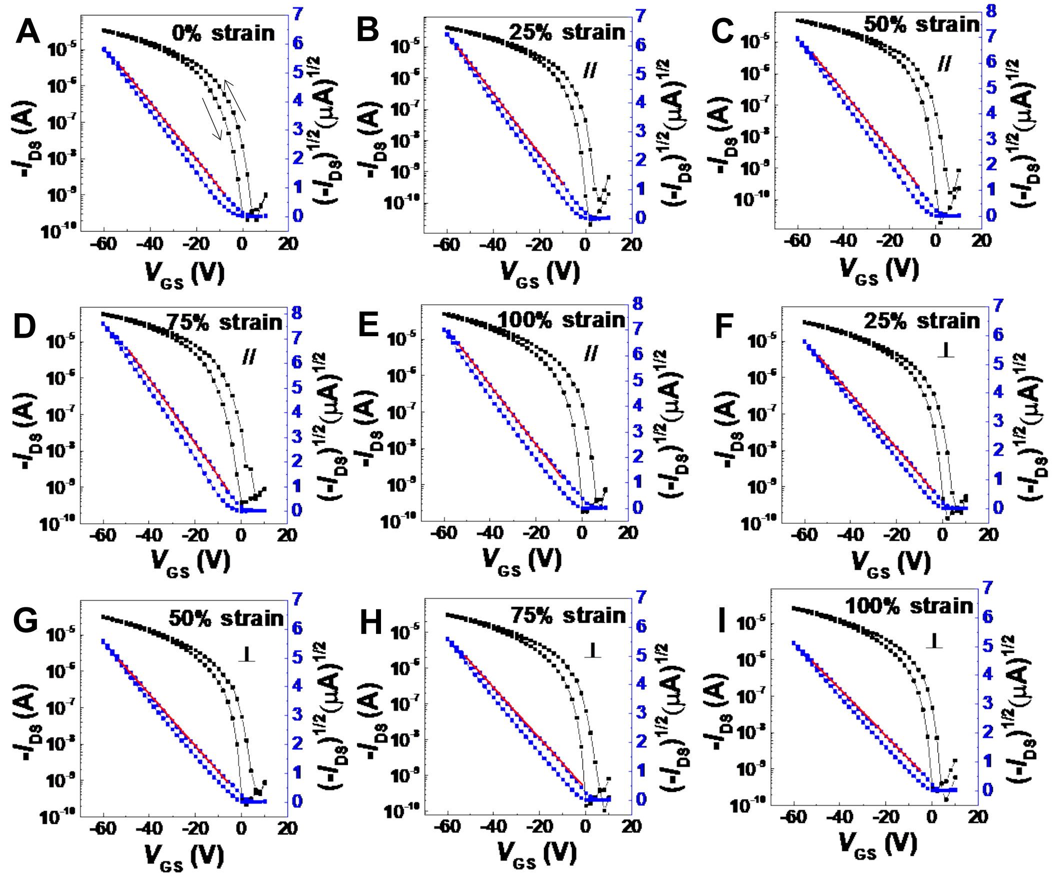

Fig. S20. Hysteresis curves (V_{DS} = −60 V) of the re-PNHDPP films under different strains during 18 months of air storage.

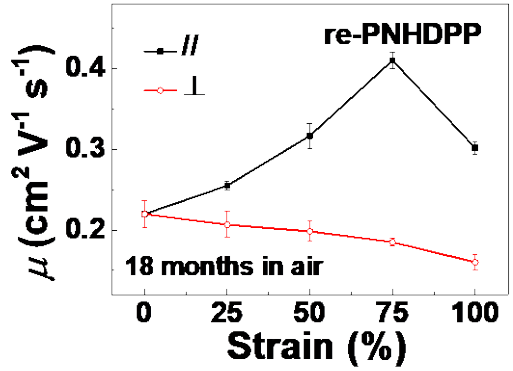

Fig. S21. Field-effect mobility as a function of various strains of re-PNHDPP films during 18 months of air storage.

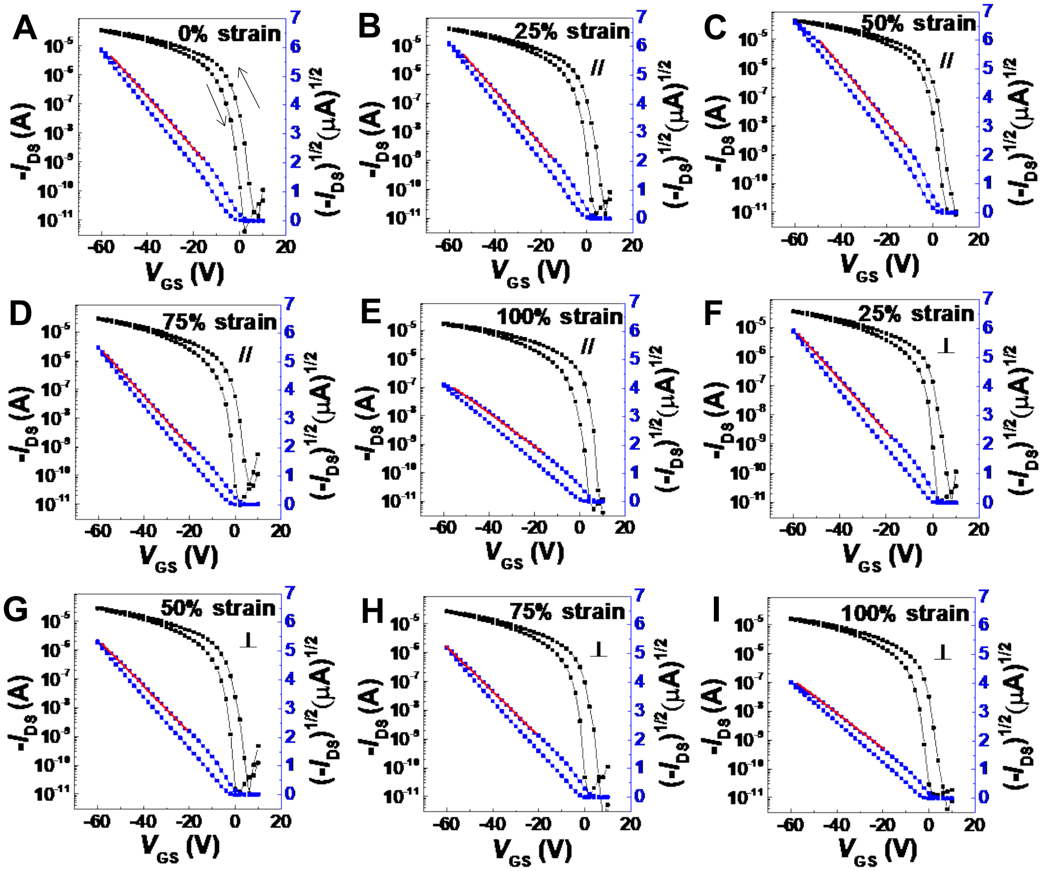

Fig. S22. **Hysteresis curves (V_{DS} = −60 V) of the ir-PNHDPP films under different strains during 18 months of air storage.**

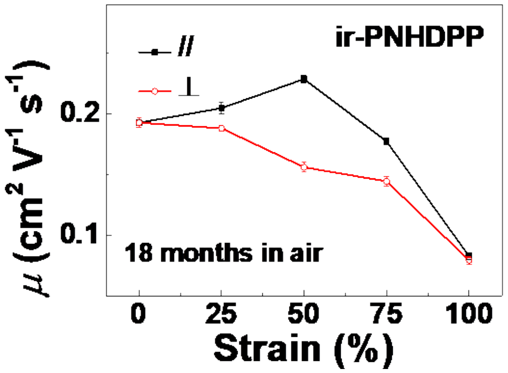

Fig. S23. **Field-effect mobility as a function of various strains of ir-PNHDPP films during 18 months of air storage.**

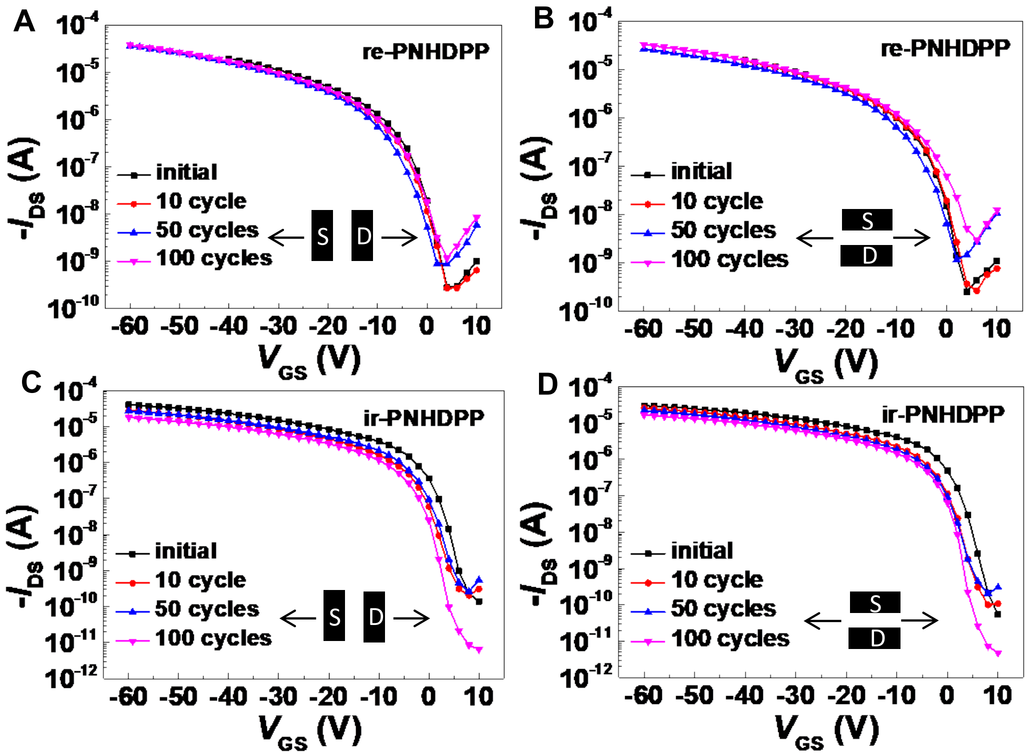

Fig. S24. Electrical characteristics of the polymer thin films at different stretch-release cycles in rigid transistor configuration. Transfer curves of the stretchable transistor based on (**A** and **B**) re-PNHDPP and (**C** and **D**) ir-PNHDPP films under 25% strain after multiple stretch-release cycles.

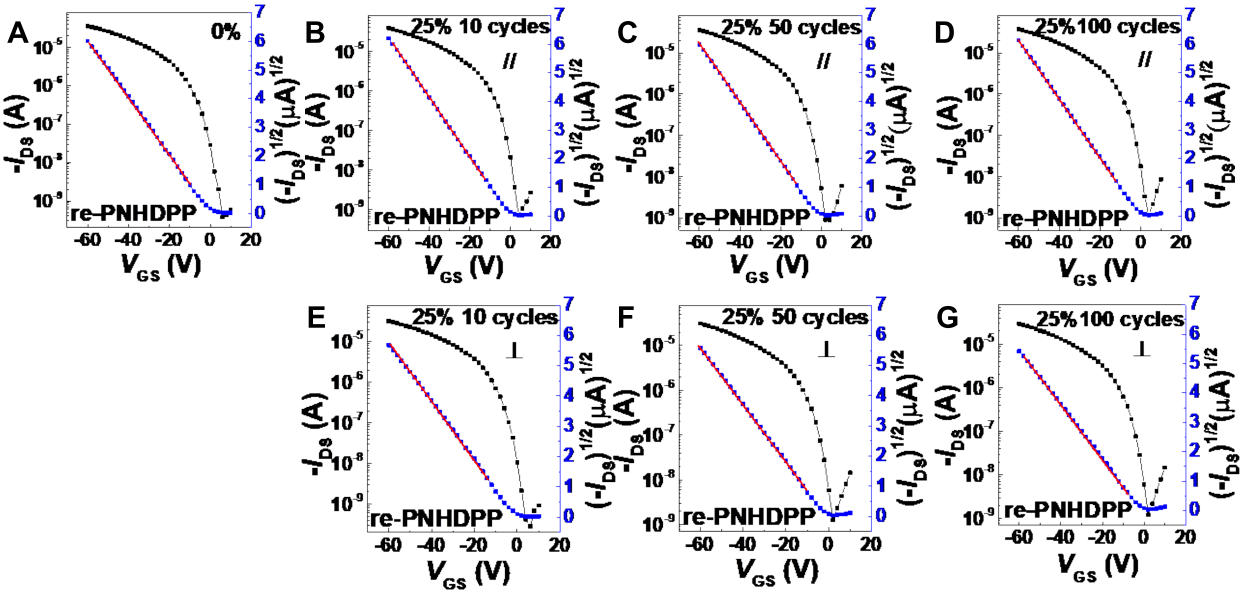

Fig. S25. Representative transfer curves of OFETs of re-PNHDPP under 25% strain for different stretching and releasing cycles and the respective plots of $(I_{DS})^{1/2}$ vs V_{GS}. Parallel represents charge transporting direction parallel to strain direction, and vertical represents charge transporting direction perpendicular to strain direction. The source-to-drain voltage is set as −60 V. The red line indicates the mobility extraction.

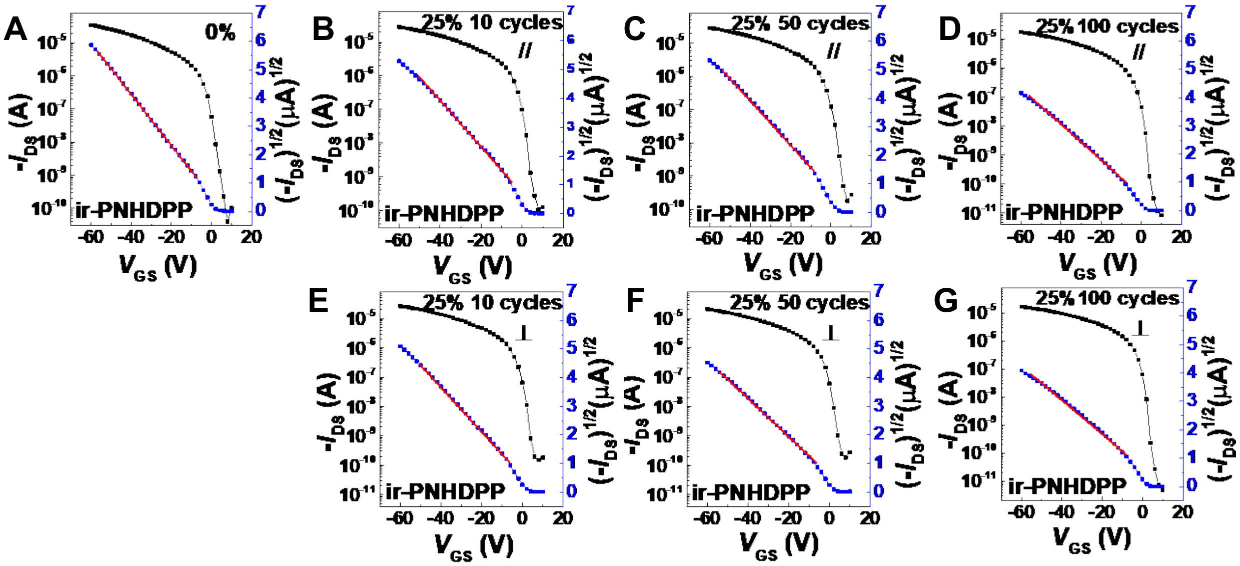

Fig. S26. **Representative transfer curves of OFETs of ir-PNHDPP under 25% strain for different stretching and releasing cycles and the respective plots of (I_{DS})$^{1/2}$ vs V_{GS}.** Parallel represents charge transporting direction parallel to strain direction, and vertical represents charge transporting direction perpendicular to strain direction. The source-to-drain voltage is set as −60 V. The red line indicates the mobility extraction.

Table S5. OFETs parameters of thin films of re-PNHDPP under 25% strain for different stretching and releasing cycles.

Cycles times	Direction	μ_{ave} (cm² V⁻¹ s⁻¹)	V_{th} (V)	I_{on}/I_{off}	*SS* (V dec⁻¹)	*r* (%)
0%		0.250 ± 0.011	0.34 ± 2.15		3.15 ± 0.25	94.36 ± 1.84
10	//	0.265 ± 0.004	-0.73 ± 0.26		2.82 ± 0.30	96.61 ± 1.05
	⊥	0.230± 0.005	-0.43 ± 0.65		2.68 ± 0.46	95.68 ± 1.05
50	//	0.272 ± 0.013	-0.67 ± 1.53	10^4-10^5	3.25 ± 0.48	94.42 ± 0.36
	⊥	0.219 ± 0.005	-1.37 ± 2.49		3.63 ± 0.71	95.85 ± 0.44
100	//	0.262 ± 0.010	0.27 ± 1.55		2.96 ± 0.48	96.83 ± 1.44
	⊥	0.203 ± 0.009	-1.85 ± 0.23		3.17 ± 0.07	93.92 ± 1.70

Table S6. OFETs parameters of thin films of ir-PNHDPP under 25% strain for different stretching and releasing cycles.

Cycles times	Direction	μ_{ave} (cm^2 V^{-1} s^{-1})	V_{th} (V)	I_{on}/I_{off}	*SS* (V dec^{-1})	*r* (%)
0%		0.206 ± 0.013	3.86 ± 1.29		2.30 ± 0.17	103.13 ± 2.59
10	//	0.173 ± 0.004	1.92 ± 0.26		2.41 ± 0.14	101.88 ± 1.56
	⊥	0.151± 0.002	1.74 ± 0.61		2.44 ± 0.04	102.24 ± 0.78
50	//	0.154 ± 0.003	1.34 ± 0.47	10^5-10^6	2.88 ± 0.12	103.25± 0.69
	⊥	0.120 ± 0.008	1.53 ± 0.42		2.85 ± 0.08	103.26 ± 1.45
100	//	0.114 ± 0.012	2.22 ± 0.68		2.03 ± 0.13	99.65 ± 2.47
	⊥	0.110 ± 0.007	1.75 ± 0.36		2.15 ± 0.19	101.13 ± 5.45

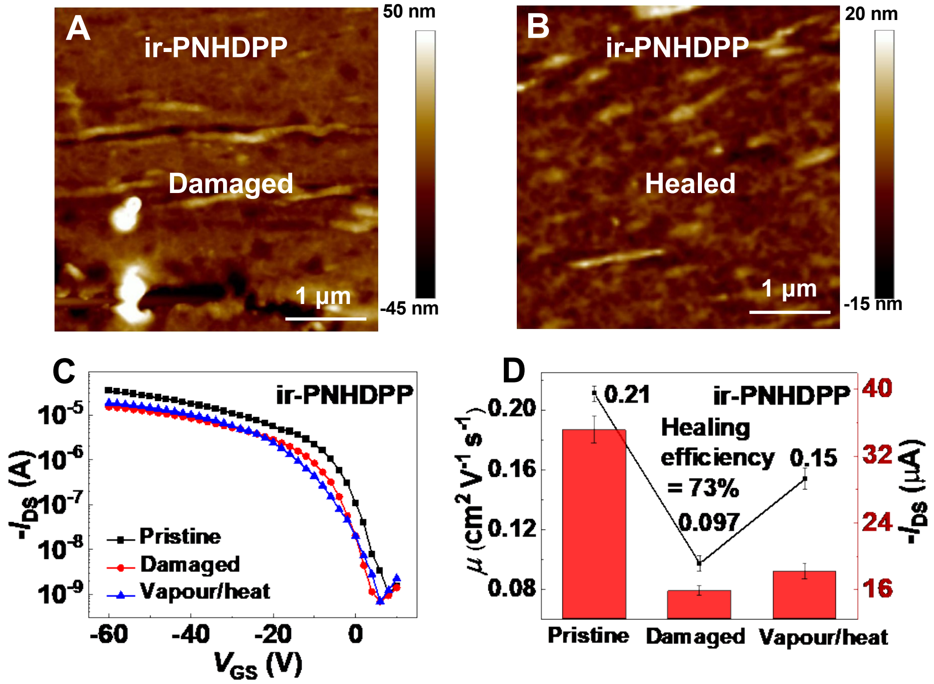

Fig. S27. Self-healing properties of ir-PNHDPP film. (**A**) AFM images for the damaged film. (**B**) AFM images for the healed film. (**C**) Transfer curves of pristine, damaged and healed ir-PNHDPP film. (**D**) Field-effect mobility and on-current of pristine, damaged and healed of ir-PNHDPP film

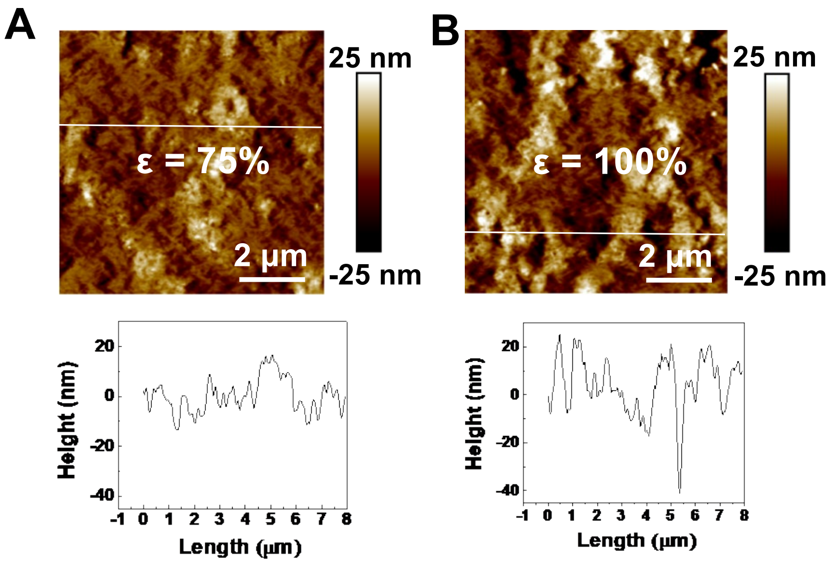

Fig. S28. **The morphology of re-PNHDPP films under different tensile strains.** AFM images and the corresponding height profiles (at the respective white lines) of re-PNHDPP under different tensile strains of 75% (**A**) and 100% (**B**).

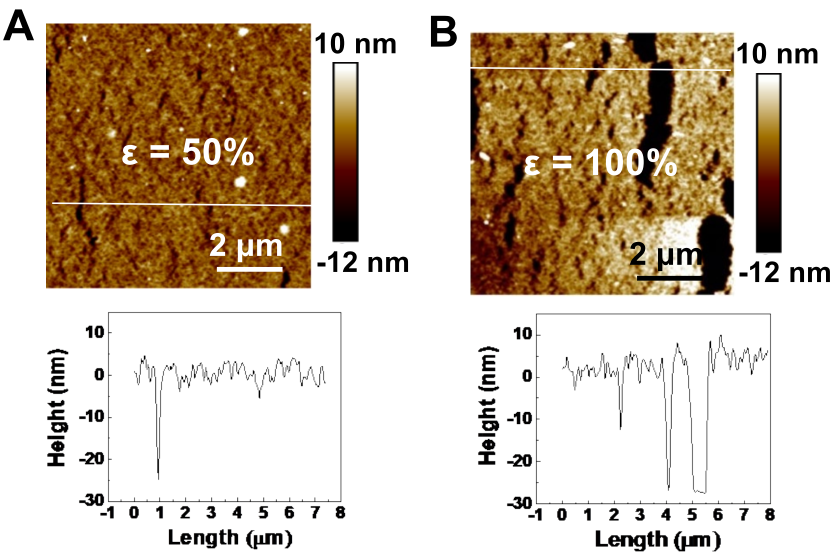

Fig. S29. The morphology of ir-PNHDPP films under different tensile strains. AFM images and the corresponding height profiles (at the respective white lines) of ir-PNHDPP under different tensile strains of 50% (**A**) and 100% (**B**).

4. Controlled film microstructures by introducing hydrogen bonds in polymer backbone

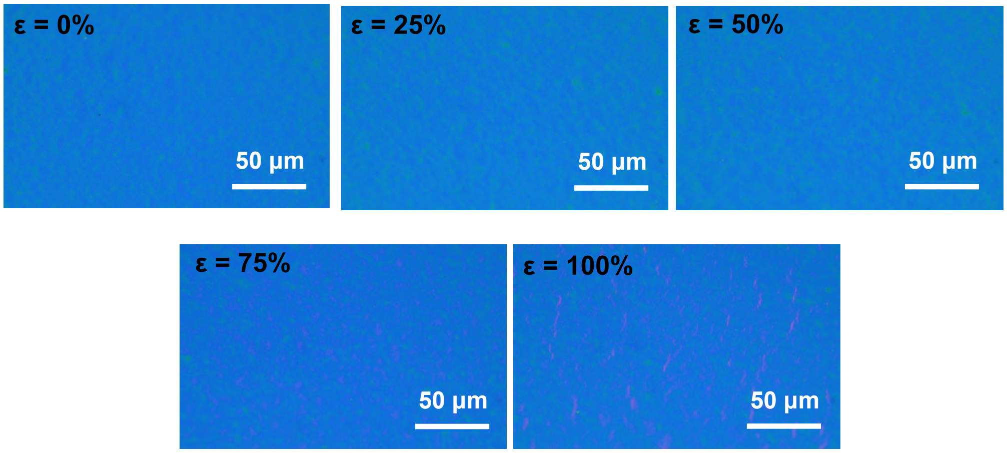

Fig. S30. Optical microscopic images of re-PNHDPP films under different strains. (Scale bar: 50 μm)

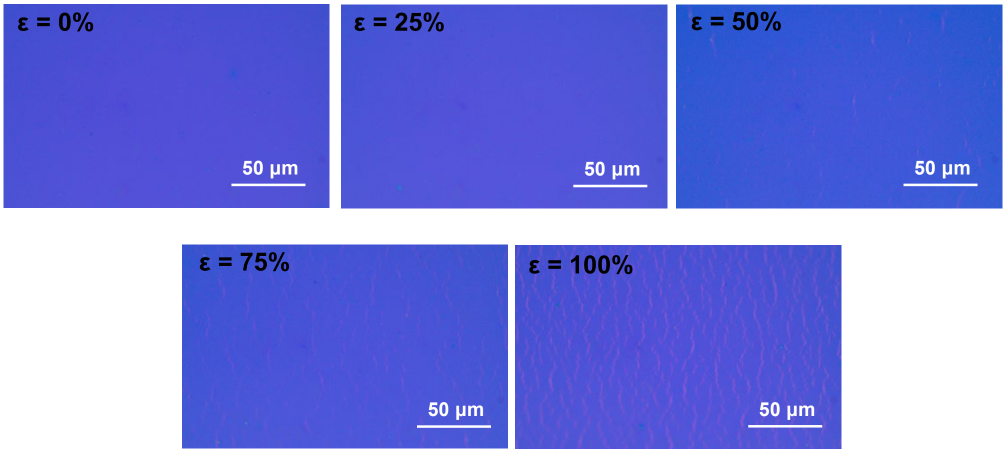

Fig. S31. Optical microscopic images of ir-PNHDPP films under different strains. (Scale bar: 50 μm)

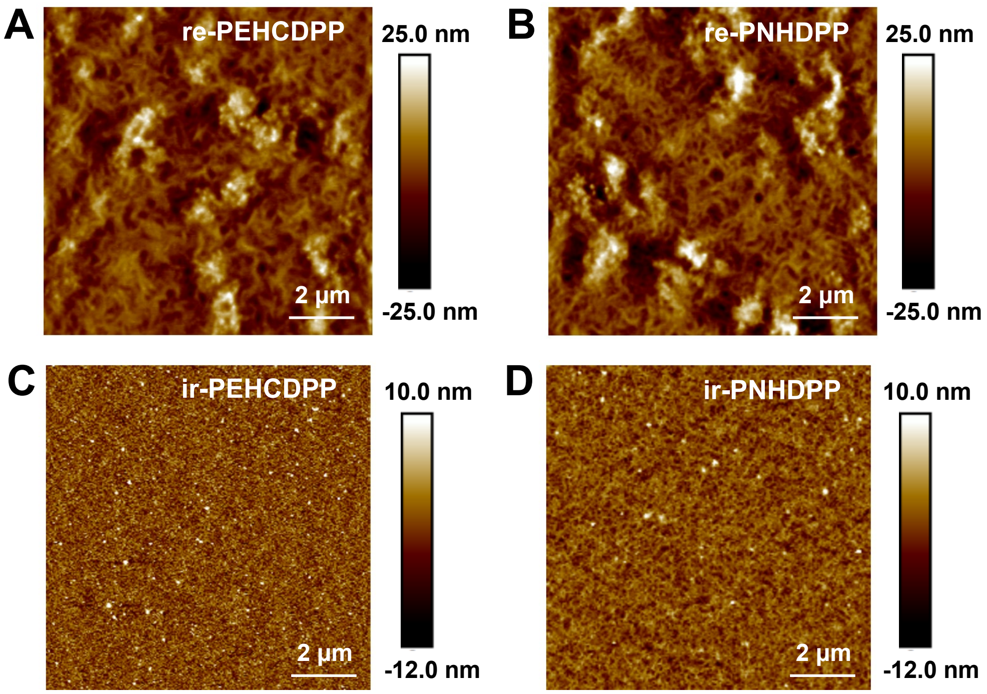

Fig. S32. The morphological characterization by AFM under the tapping mode. (**A**) The height image of re-PEHCDPP. (**B**) The height image of re-PNHDPP. (**C**) The height image of ir-PEHCDPP. (**D**) The height image of ir-PNHDPP.

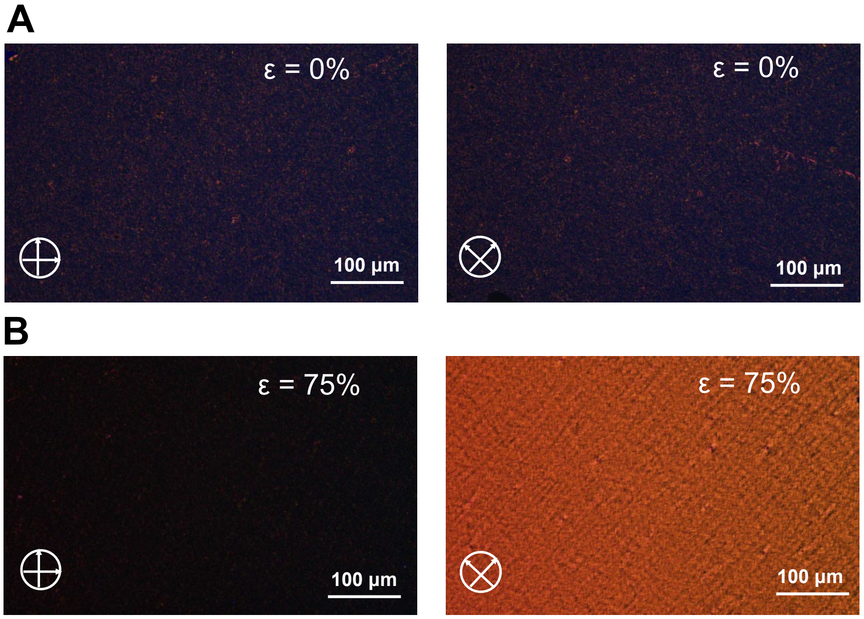

Fig. S33. **Cross-polarized optical microscopy images of re-PNHDPP film.** **(A)** 0% strain. **(B)** 75% strain. The orientation of the cross-polarizers is shown as crossed arrows. (Scale bar: 100 μm)

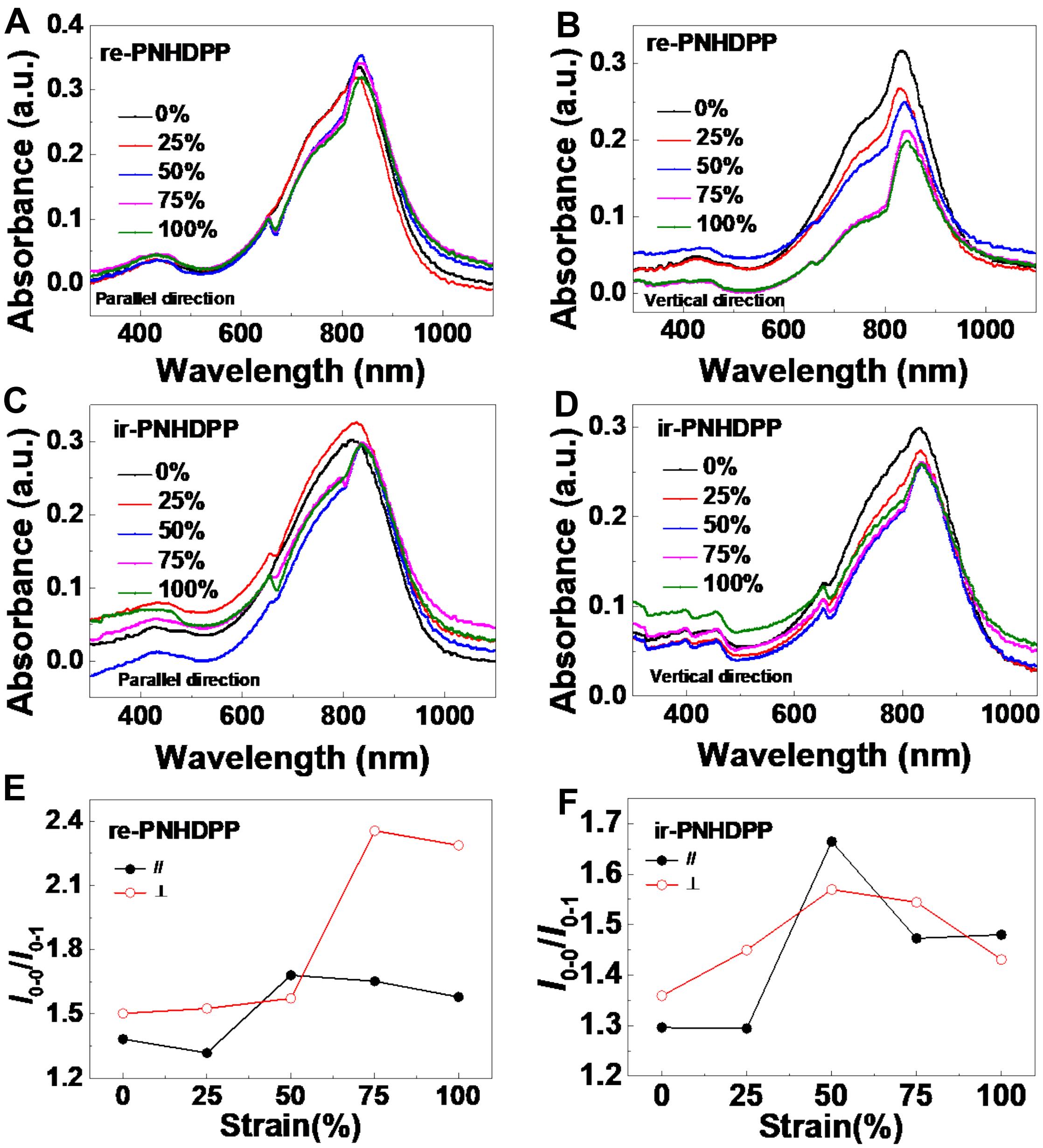

Fig. S34. **Polarized absorption properties.** Polarized absorption spectra of re-PNHDPP and ir-PNHDPP films under different strains with polarizer direction parallel to the strain direction (**A, C**) and perpendicular to the strain direction (**B, D**). The changes in chain aggregation of re-PNHDPP (**E**) and ir-PNHDPP (**F**) under the different strains based on the 0-0 absorption peak.

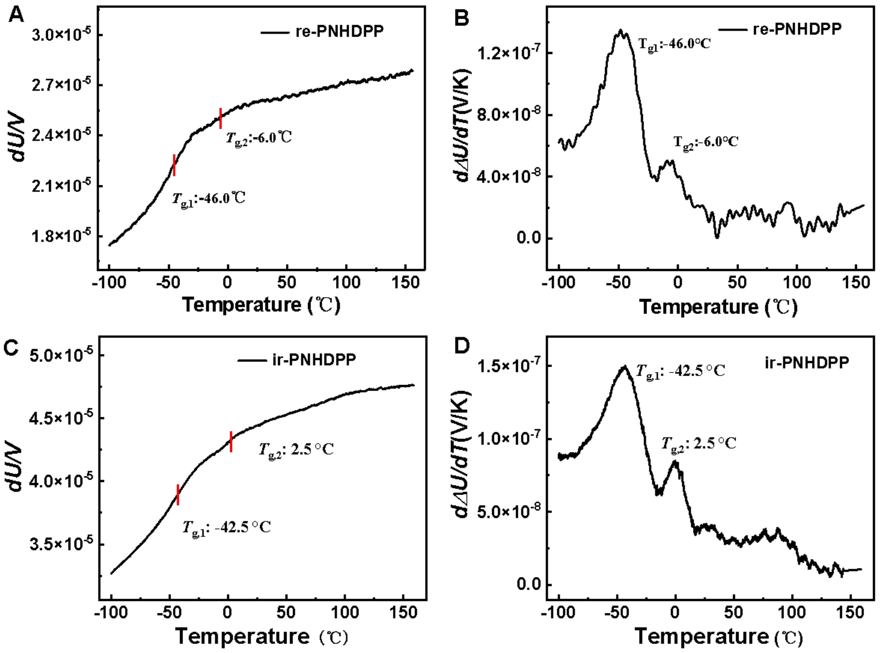

Fig. S35. **Calorimetric ac-chip measurement for the polymer films.** Heating curves **(A)** and the first derivative curves **(B)** of re-PNHDPP, and the Heating curves **(C)** and the first derivative curves **(D)** of ir-PNHDPP, which give the glass transition temperatures.

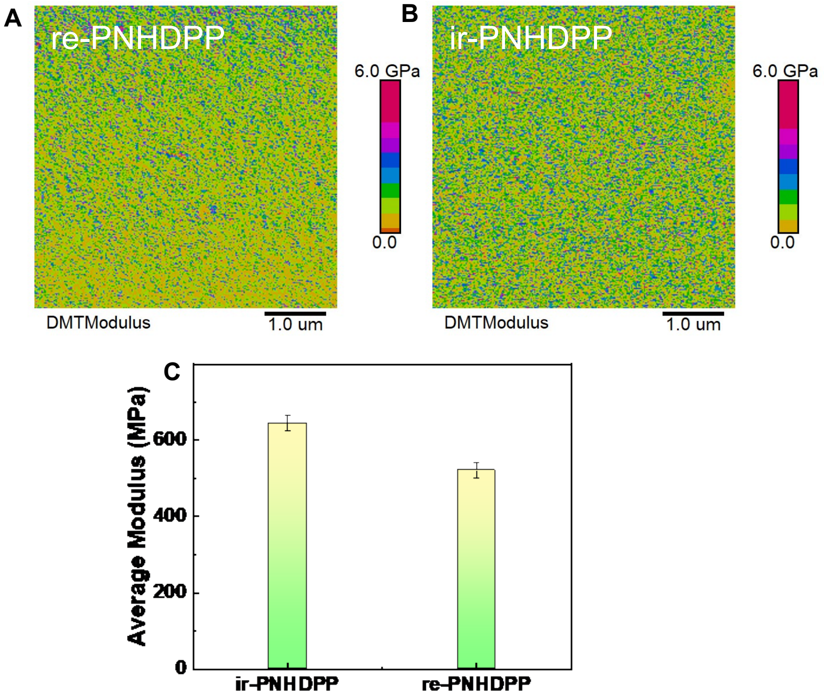

Fig. S36. **The tensile moduli by AFM nanomechanical mapping. (A)** re-PNHDPP**. (B)** ir-PNHDPP. (**C**) The average modulus measurement results for two polymer films.

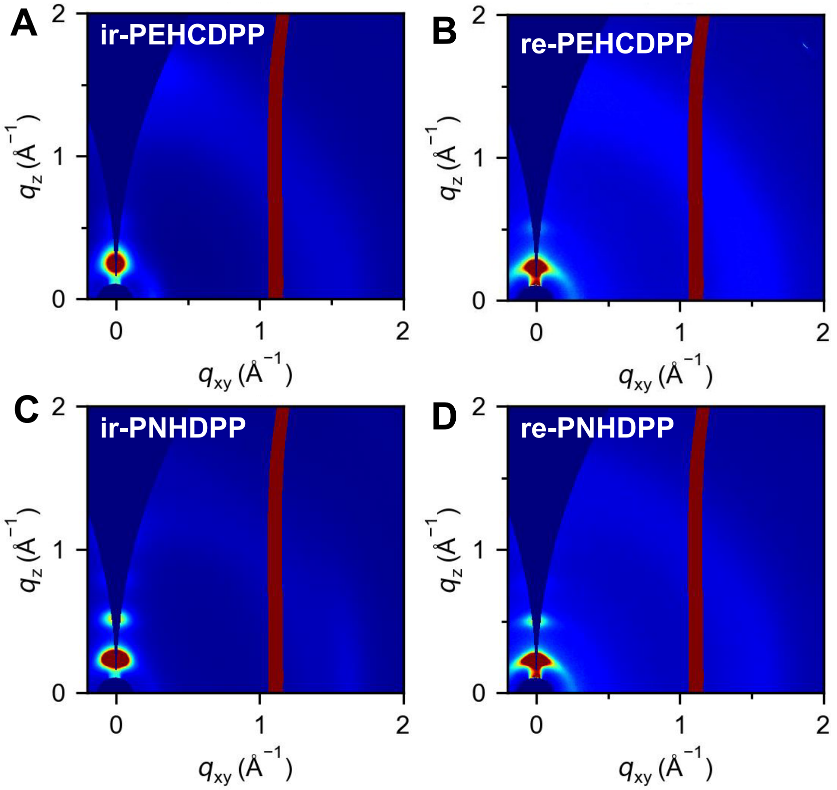

Fig. S37. **The GIWAXS of polymer films before and after thermal annealing**. (**A**) ir-PEHCDPP. (**B**), re-PEHCDPP. (**C**) ir-PNHDPP. (**D**) re-PNHDPP

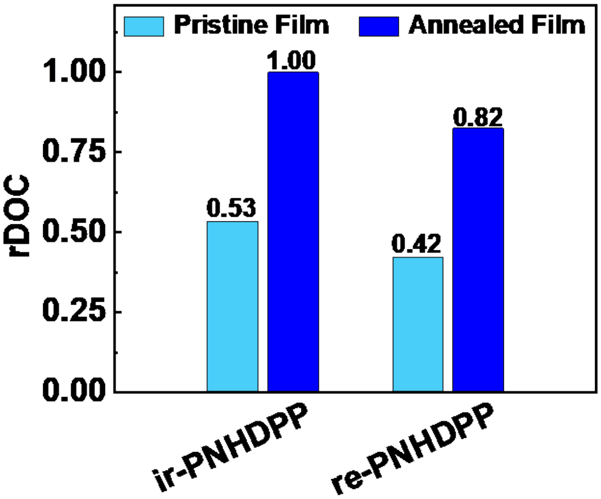

Fig. S38. **The changes of relative degree of crystallinity (rDoC) extracted from peak (200) of polymer before and after thermal annealing.**

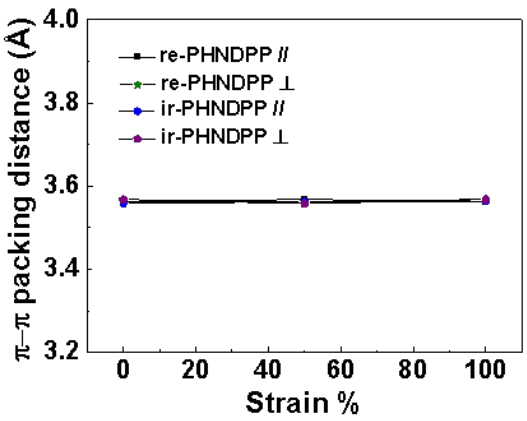

Fig. S39. **The π-π stacking distances of re-PNHDPP and ir-PNHDPP films under different stretching strains.**

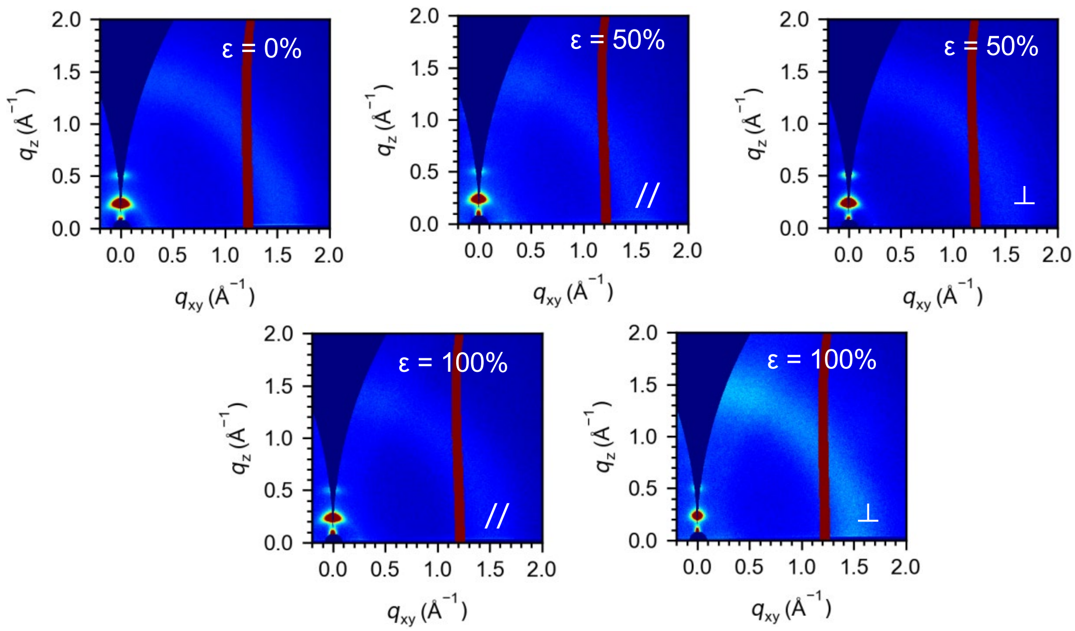

Fig. S40. **GIWAXS of re-PNHDPP films.** The results were obtained under different strains along the parallel or perpendicular direction of incident X-ray with respect to the strain.

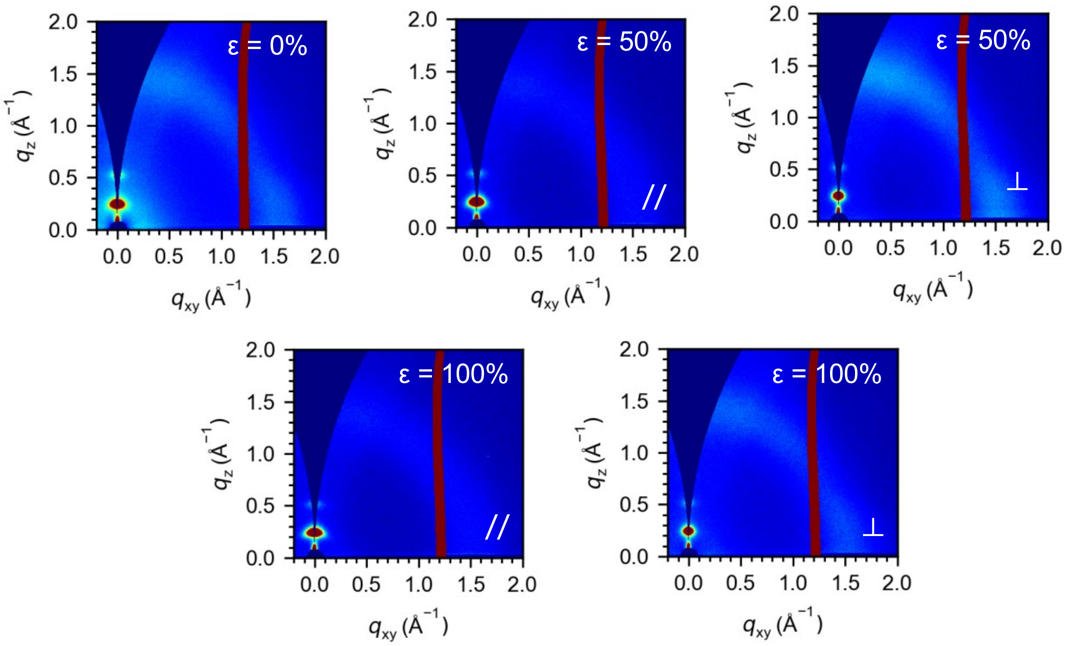

Fig. S41. **GIWAXS of ir-PNHDPP films.** The results were obtained under different strains along the parallel or perpendicular direction of incident X-ray with respect to the strain.

Table S7. Structural parameters of re-PNHDPP and ir-PNHDPP films extracted from the GIWAXS patterns.

	r-DOC (200)[a]	Lamellar fwhm [Å^{-1}][b]	CL [Å][c]	Lamellar spacing [Å][d]
re-PNHDPP	0.82	0.0457	123.5	25.90
Ir-PNHDPP	1	0.0510	110.8	24.87

[a] Determined by element analyzer. Extracted from the (200) lamellar stacking peak. [b] Extracted from (100) diffraction peak. [c] CL of (100) peak calculated with 2πk/fwhm, where k is a shape factor (k = 0.9). [d] Extracted from (100) diffraction peak, the d-spacing was calculated by Bragg equation ($d = 2\pi/q$).

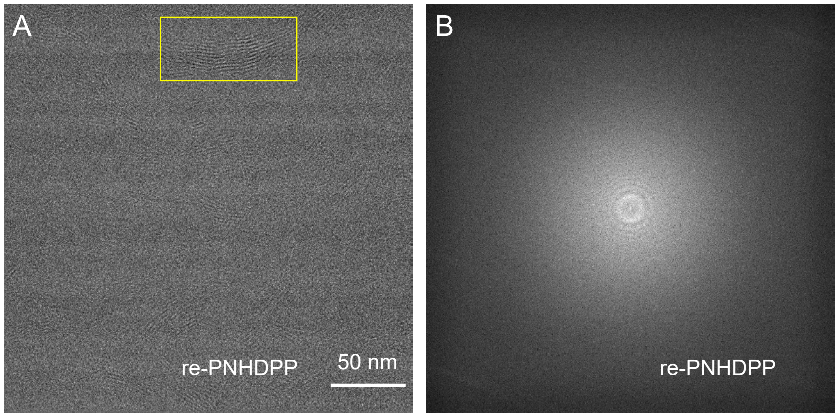

Fig. S42. **The cryo-TEM data of re-PNHDPP.** (**A**) Cryo-TEM image. (**B**) The representative fast fourier transform (FFT).

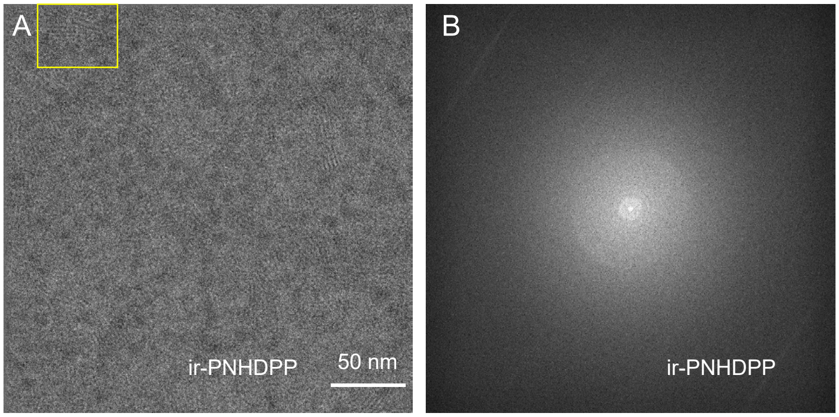

Fig. S43. **The cryo-TEM data of ir-PNHDPP.** (**A**) Cryo-TEM image. (**B**) The representative fast fourier transform (FFT).

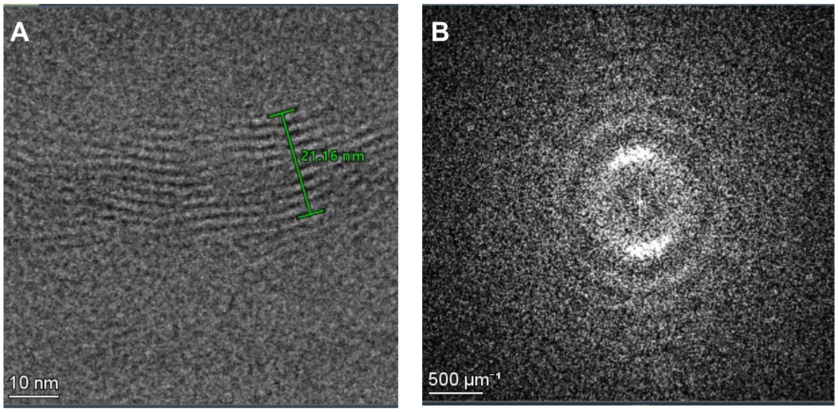

Fig. S44. The enlarged cryo-TEM data of re-PNHDPP. (**A**) Cryo-TEM image. (**B**) The representative fast fourier transform (FFT).

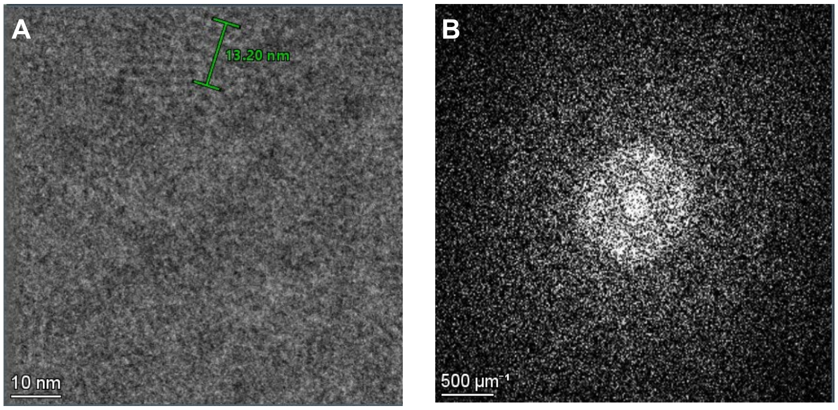

Fig. S45. The enlarged cryo-TEM data of ir-PNHDPP. (**A**) Cryo-TEM image. (**B**) The representative fast fourier transform (FFT).

5. Further optimization of regular polymer and fabrication of fully stretchable transistors

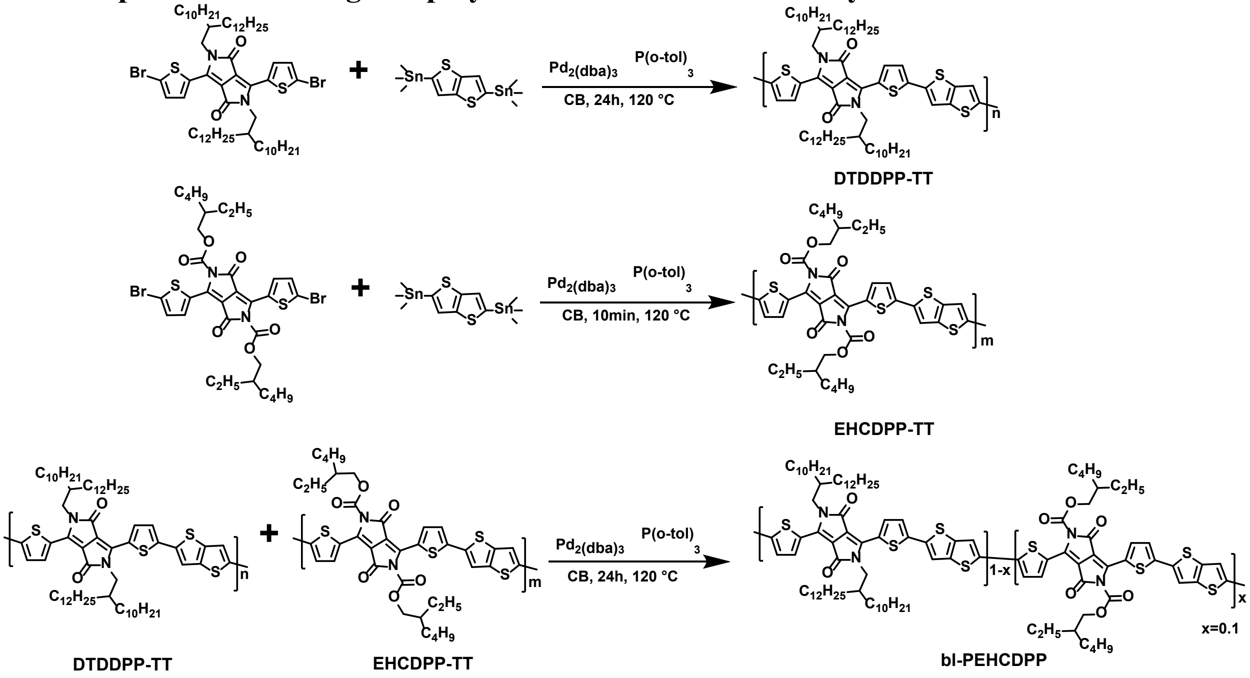

Fig. S46. **Synthetic route of block polymer bl-PEHCDPP.**

Synthesis of block polymer bl-PEHCDPP: The synthesis procedure of block polymer bl-PEHCDPP is similar to that of re-PEHCDPP. The whole synthesis process is mainly divided into two steps for bl-PEHCDPP. Firstly, 2,5-bis(trimethylstannyl)thieno[3,2-b]thiophene (TT, 83.9 mg, 0.18 mmol), 3,6-bis-(5-bromo-thiophen-2-yl)-2,5-bis(2-decyltetradecyl)-1,4-dioxo-pyrrolo[3,4-c]pyrrole (DPP, 203.6 mg, 0.18 mmol), $Pd_2(dba)_3$ (5.4 mg), $P(o\text{-}tol)_3$ (14.7 mg), and dry chlorobenzene (3.5 mL) were added to a 50 mL fused Schlenk tube, which was charged with nitrogen through a freeze-pump-thaw cycle for three times. The reaction mixture was stirred for 24 h at 120°C under nitrogen atmosphere. Secondly, 2,5-bis(trimethylstannyl)thieno[3,2-b]thiophene (TT, 9.3 mg, 0.02 mmol), 3,6-bis(5-bromo-thiophen-2-yl)-2,5-bis(2-ethylhexyl-carboxylate)-1,4-dioxo-pyrrolo[3,4-c]pyrrole (EHC-DPP, 15.6 g, 0.02 mmol), $Pd_2(dba)_3$ (0.6 mg), $P(o\text{-}tol)_3$ (1.6 mg), and dry chlorobenzene (1.5 mL) were added to a 10 mL fused Schlenk tube, which was charged with nitrogen through a freeze-pump-thaw cycle for three times. Next, the reaction mixture was heated at 120°C for 10 min, then quickly transferred to the first reaction system (50mL reaction bottle) and stirred for 24 h at 120°C under nitrogen atmosphere. The detailed post-processing procedure is similar to that of ir-PEHCDPP and finally obtained a glossy film after removing the solvent (134 mg, 62%). GPC: M_n = 40.6 kDa, M_w = 118.9 kDa, PDI = 2.93.

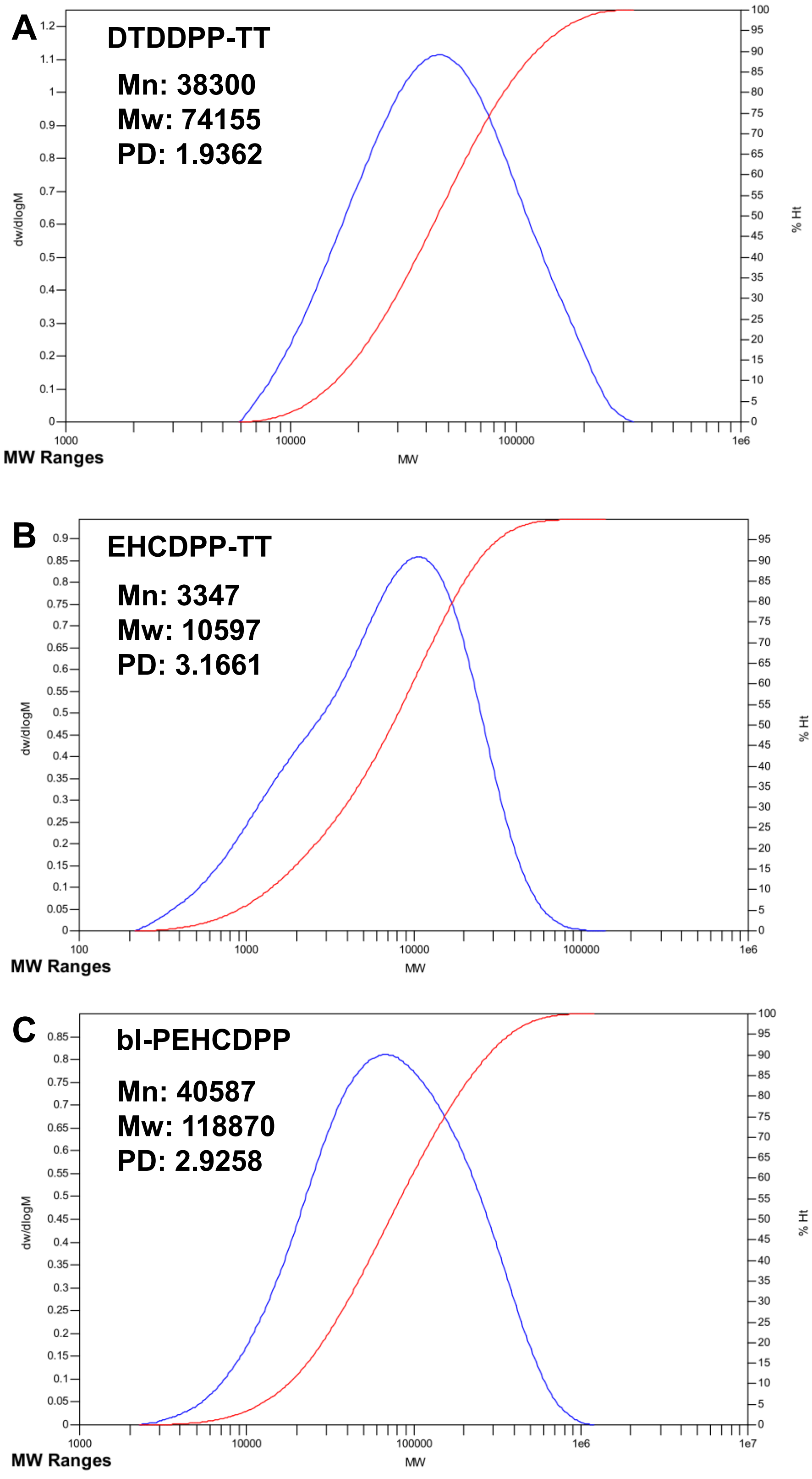

Fig. S47. **The GPC data.** (**A**) DTDDPP-TT. (**B**) EHCDPP-TT. (**C**) bl-PEHCDPP polymer.

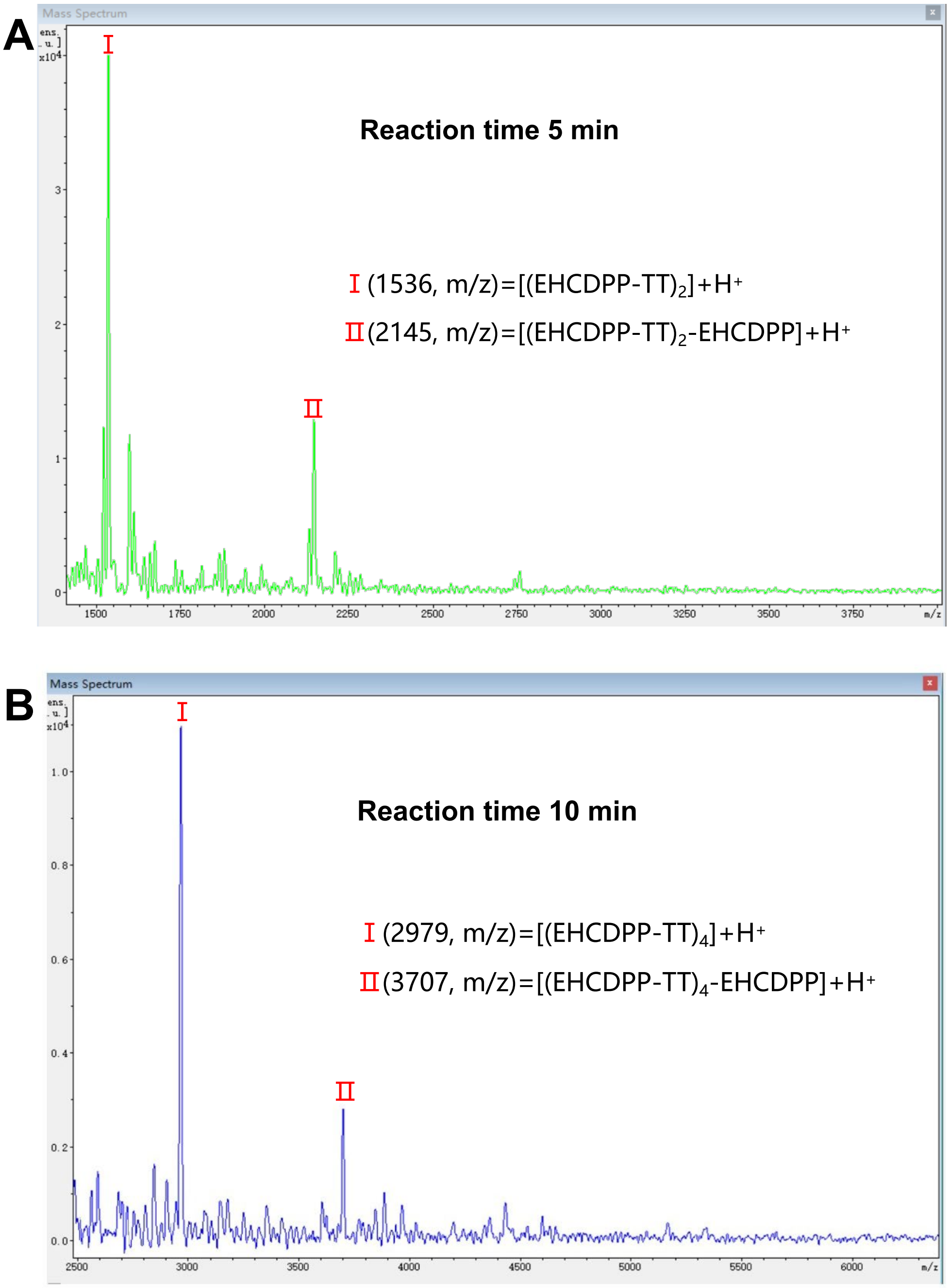

Fig. S48. **MALDI-TOF mass data for the prepolymer EHCDPP-TT with different reaction time.** (**A**) 5 min reaction time. (**B**) 10 min reaction time.

Table S8. Elemental analysis of polymers before and after thermal annealing

Composite	C	H	N	S	Average ratio of EHC or NH group[a]	Data sources
PEHCDPP	72.09%	9.18%	2.66%	12.15%	10.00%	Theoretical[b]
PNHDPP	71.59%	8.88%	2.91%	13.30%	10.00%	Theoretical[c]
bl-PEHCDPP	72.81%	9.22%	2.64%	12.10%	9.24%	Measured[d]
bl-PNHDPP	71.72%	8.95%	2.89%	13.14%	9.15%	Measured[d]

[a)] Derived from the measured C, H, N, S element contents and calculated based on the theoretical ratio respectively. [b)] Derived from 10% part NHDPP-TT and 90% part DTDDPP-TT. [c)] Derived from 10% part NHDPP-TT and 90% part DTDDPP-TT. [d)] Determined by element analyzer.

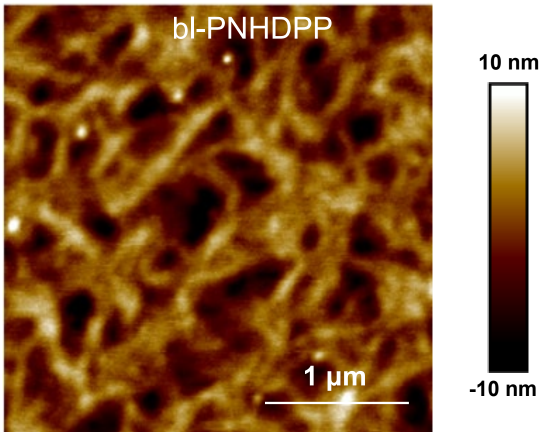

Fig. S49. AFM images for the film of bl-PNHDPP.

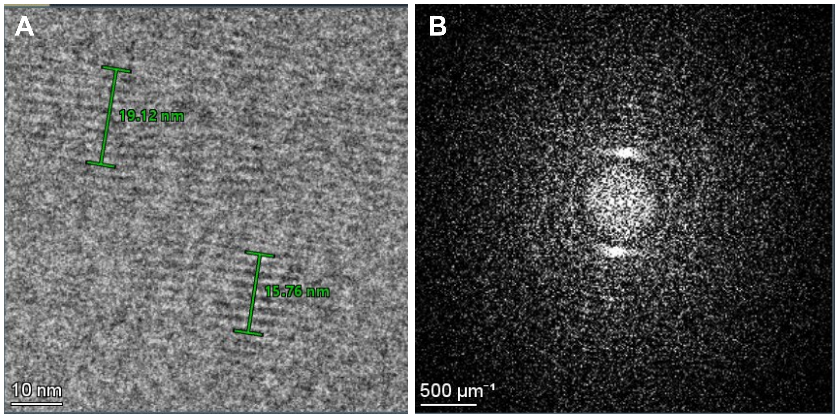

Fig. S50. The cryo-TEM data of bl-PNHDPP. (**A**) Cryo-TEM image. (**B**) The representative fast fourier transform (FFT).

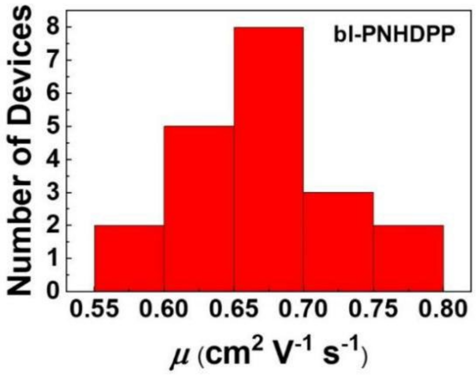

Fig. S51. Distribution of the field-effect mobilities for bl-PNHDPP film.

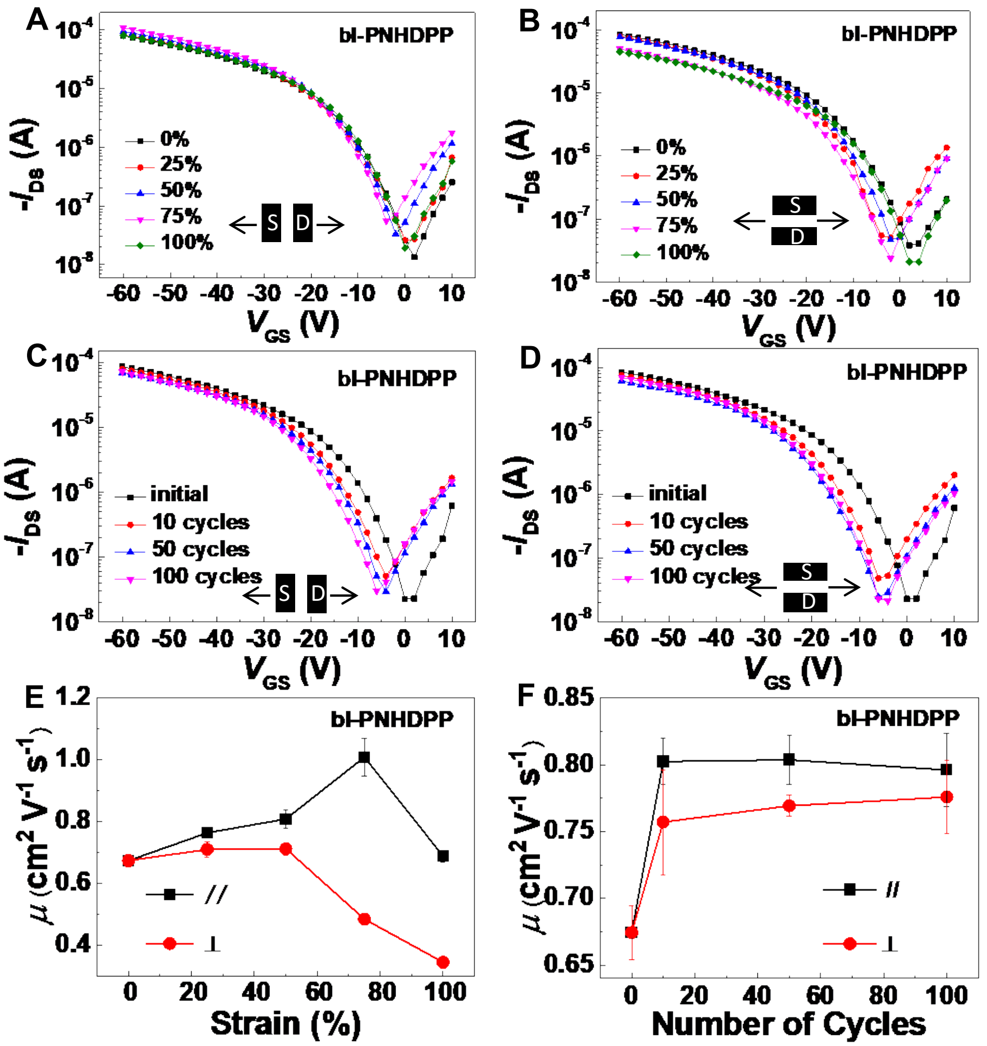

Fig. S52. Electrical characteristics of bl-PNHDPP films at strains. Transfer curves of at different strains along the charge transport direction (**A**) and perpendicular to charge transport direction (**B**). Transfer curves of bl-PNHDPP films under 25% strain after multiple stretch-release cycles along the charge transport direction (**C**) and perpendicular to charge transport direction (**D**). (**E**) Field-effect mobilities as a function of various strains. (**F**) Field-effect mobilities versus number of stretching cycles.

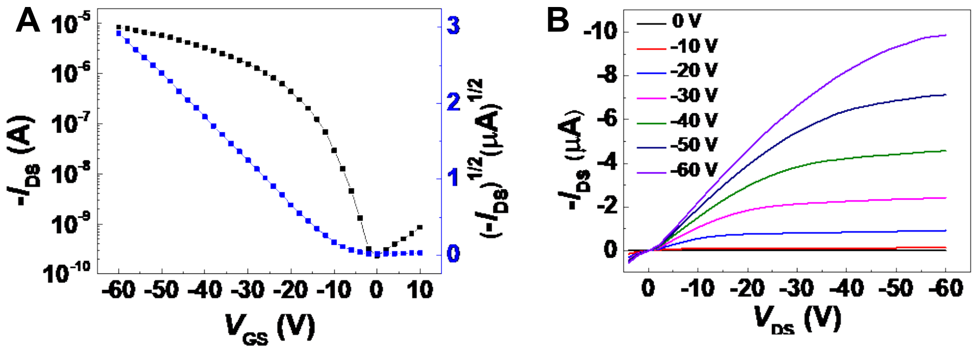

Fig. S53. A typical transfer curve (A) and output curves (B) of a fully stretchable transistor with bl-PNHDPP film at 0% strain.

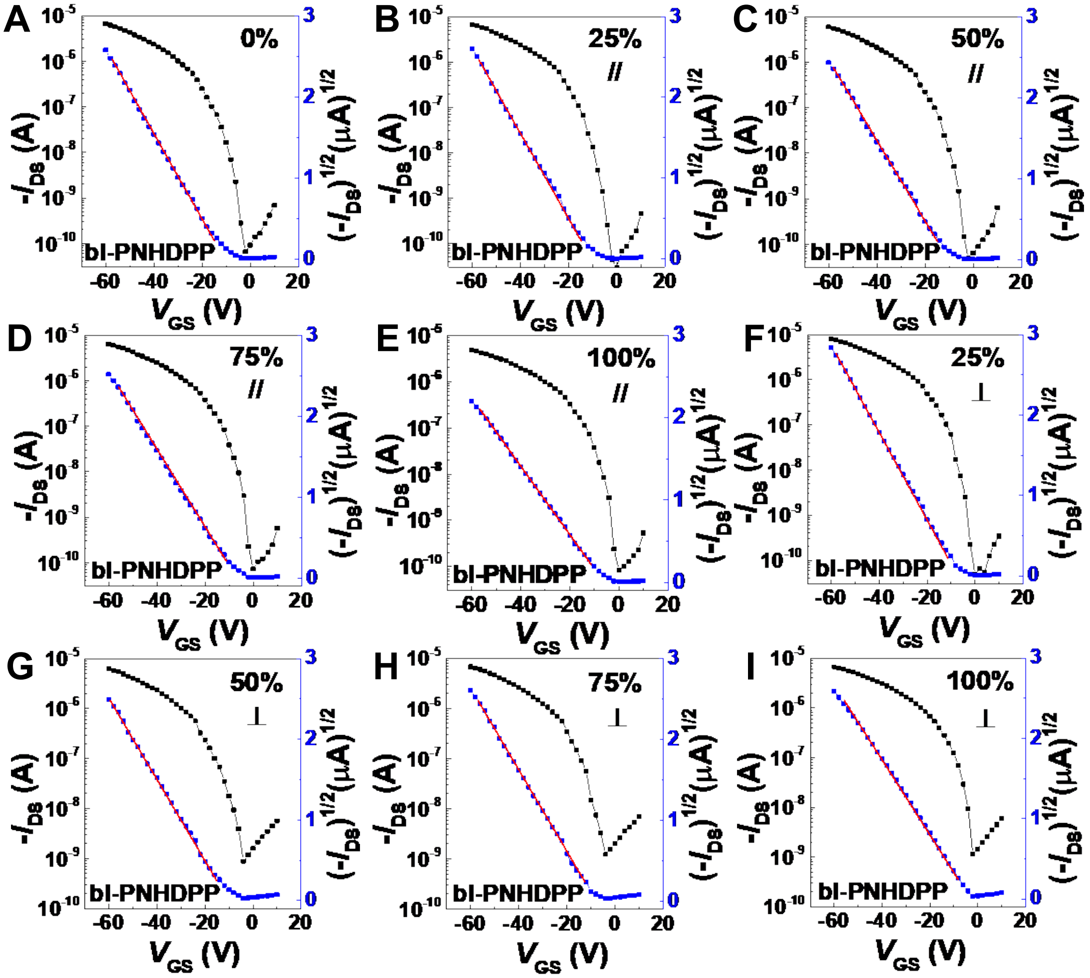

Fig. S54. **Representative transfer curves of fully stretchable organic transistors of bl-PNHDPP under different strains and the respective plots of (I_{DS})$^{1/2}$ vs V_{GS}.** Parallel represents charge transporting direction parallel to strain direction, and vertical represents charge transporting direction perpendicular to strain direction. The source-to-drain voltage is set as −60 V. The red line indicates the mobility extraction.

Table S9. OFETs parameters of thin films of bl-PNHDPP under different strains.

Strain	Direction	μ_{ave} (cm^2 V^{-1} s^{-1})	V_{th} (V)	I_{on}/I_{off}	*SS* (V dec^{-1})	*r* (%)
0%		0.891 ± 0.065	-9.38 ± 1.42		3.77 ± 0.92	81.35 ± 2.78
25%	//	1.105 ± 0.033	-10.5 ± 1.32	10^4-10^5	3.64 ± 1.12	80.92 ± 1.71
	⊥	0.589± 0.025	-9.02 ± 1.73		3.27 ± 0.08	81.98 ± 2.79
50%	//	1.113 ± 0.055	-8.83 ± 2.84		4.61 ± 1.85	83.06 ± 4.21
	⊥	0.399 ± 0.009	-8.93 ± 0.41	10^3-10^4	4.83 ± 0.37	82.76 ± 0.87
75%	//	1.273 ± 0.031	-9.67 ± 1.53	10^4-10^5	3.50 ± 0.41	80.91 ± 1.72
	⊥	0.270 ± 0.008	-7.21 ± 1.04	10^3-10^4	3.22 ± 0.23	86.43 ± 1.07
100%	//	1.080 ± 0.026	-8.16 ± 2.56	10^4-10^5	4.27 ± 0.83	82.45 ± 3.13
	⊥	0.164 ± 0.002	-3.83 ± 0.76	10^3-10^4	3.46 ± 0.23	89.33 ± 2.08

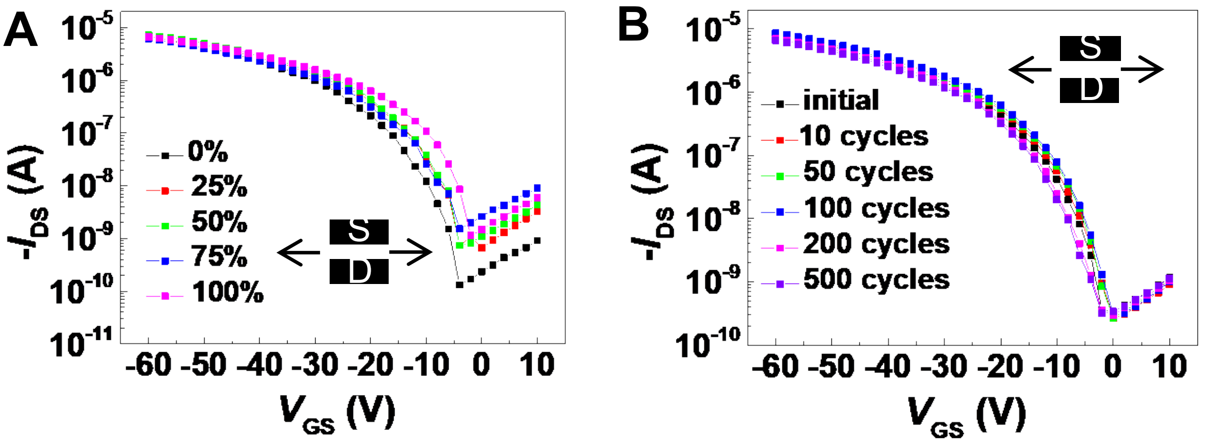

Fig. S55. **Electrical characteristics of fully stretchable transistors based on bl-PNHDPP.** (**A**) Transfer curves of the stretchable transistor with bl-PNHDPP film at different strains perpendicular to charge transport direction. (**B**) Transfer curves of the stretchable transistor with bl-PNHDPP film under 25% strain after multiple stretch-release cycles perpendicular to charge transport direction.

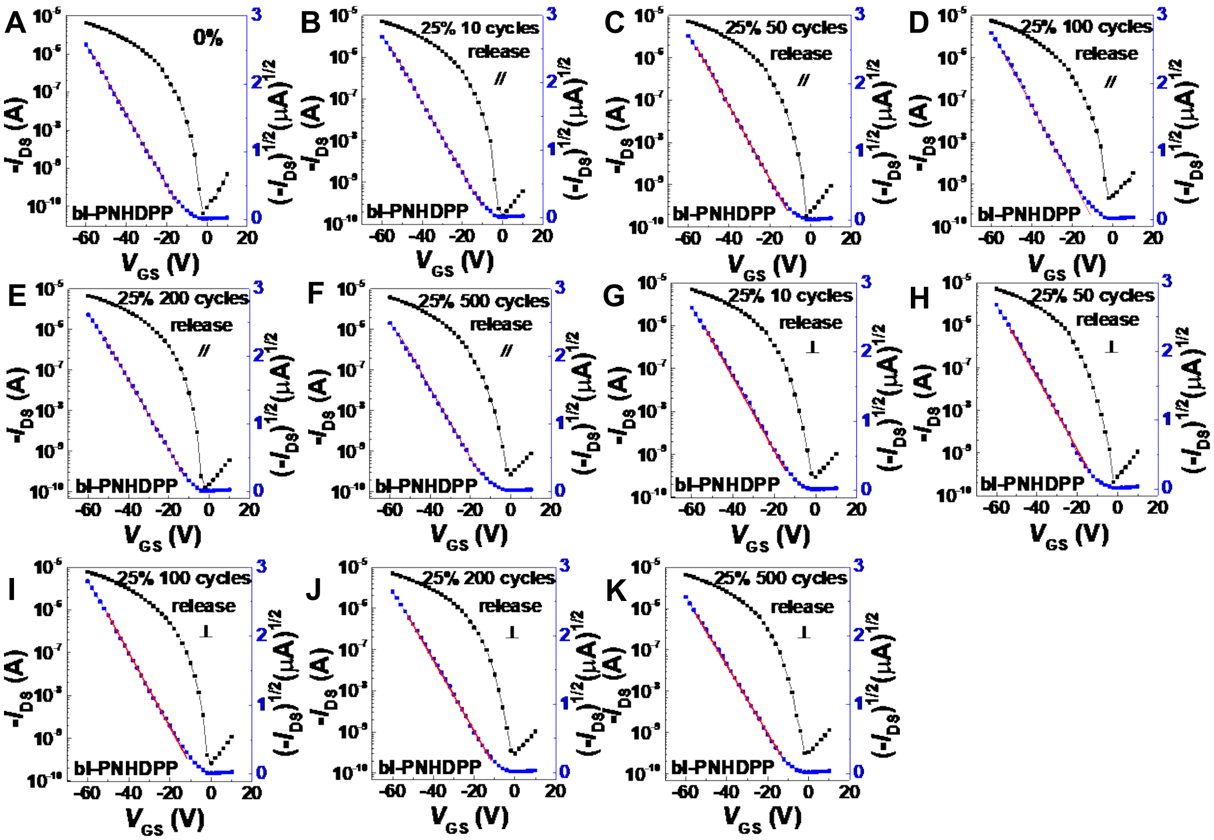

Fig. S56. Representative transfer curves of fully stretchable organic transistors of bl-PNHDPP under 25% strain for different stretching and releasing cycles and the respective plots of (I_{DS})$^{1/2}$ vs V_{GS}. Parallel represents charge transporting direction parallel to strain direction, and vertical represents charge transporting direction perpendicular to strain direction. The source-to-drain voltage is set as −60 V. The red line indicates the mobility extraction.

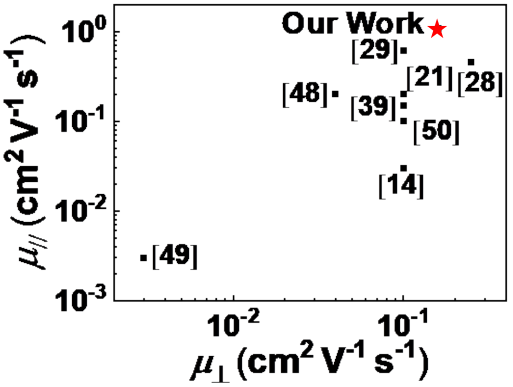

Fig. S57. The mobilities of fully stretchable bl-PNHDPP device in two directions under 100% strain compared with those of intrinsically stretchable polymers reported in the literatures.

Table S10. OFETs parameters of thin films of bl-PNHDPP under 25% strain for different stretching and releasing cycles.

Cycles times	Direction	μ_{ave} ($cm^2 V^{-1} s^{-1}$)	V_{th} (V)	I_{on}/I_{off}	*SS* ($V dec^{-1}$)	*r* (%)
0%		0.891 ± 0.065	-9.38 ± 1.42	10^4-10^5	3.77 ± 0.92	81.35 ± 2.78
10	//	0. 887± 0.048	-8.12 ± 1.84		3.26 ± 0.09	82.06 ± 1.01
	⊥	0.903± 0.034	-8.84 ± 1.80		4.29 ± 0.88	82.56 ± 4.41
50	//	0.893 ± 0.036	-9.02 ± 1.27		3.52 ± 0.32	82.24 ± 2.37
	⊥	0.899 ± 0.034	-8.82 ± 2.74		4.05± 0.49	85.44 ± 1.98
100	//	0.890 ± 0.027	-8.53 ± 1.58		3.74± 0.49	83.31 ± 1.87
	⊥	0.886 ± 0.013	-6.86 ± 0.77		4.16 ± 0.51	86.62 ± 3.08
200	//	0.866 ± 0.011	-7.77 ± 0.274		4.62 ± 0.49	83.37 ± 1.87
	⊥	0.862 ± 0.012	-7.54 ± 0.45		4.24 ± 0.63	84.65 ± 2.28
500	//	0.841 ± 0.017	-9.06 ± 2.09		3.96 ± 0.12	82.34 ± 4.89
	⊥	0.820 ± 0.018	-6.86 ± 1.30		3.99 ± 0.34	85.38 ± 2.30

Table S11. Device geometry and dielectric capacitance change as function of strain in the fully stretchable transistor.

Stretching direction	Strain (%)	Channel length (μm)	Channel width (μm)	Capacitance (nF/cm^2)
parallel	0	200	1000	1.13
	25	250	930	1.45
	50	300	860	1.57
	75	350	790	1.68
	100	400	720	1.85
vertical	0	200	1000	1.13
	25	186	1250	1.45
	50	172	1500	1.57
	75	158	1750	1.68
	100	144	2000	1.85
